# Histone H3K27 methyltransferase EZH2 and demethylase JMJD3 regulate hepatic stellate cells activation and liver fibrosis

**DOI:** 10.7150/thno.46360

**Published:** 2021-01-01

**Authors:** Yan Jiang, Chan Xiang, Fan Zhong, Yang Zhang, Liyan Wang, Yuanyuan Zhao, Jiucun Wang, Chen Ding, Li Jin, Fuchu He, Haijian Wang

**Affiliations:** 1 Shanghai Fifth People's Hospital of Fudan University, Shanghai Key Laboratory of Medical Epigenetics, Ministry of Science and Technology International Co-Laboratory of Medical Epigenetics and Metabolism, Institutes of Biomedical Sciences, Shanghai Medical College, Fudan University, Shanghai, China.; 2 Center for Medical Research and Innovation, Shanghai Pudong Hospital and Pudong Medical Center, Shanghai Medical College, Fudan University, Shanghai, China.; 3 State Key Laboratory of Genetic Engineering and Collaborative Innovation Center for Genetics and Development, Ministry of Education Key Laboratory of Contemporary Anthropology and Department of Anthropology and Human Genetics, School of Life Sciences, Fudan University, Shanghai, China.; 4 State Key Laboratory of Proteomics, Beijing Proteome Research Center, National Center for Protein Sciences, Beijing Institute of Lifeomics, Beijing, China.

**Keywords:** liver fibrosis, HSC, EZH2, JMJD3, epigenetics

## Abstract

**Rationale:** As the central hallmark of liver fibrosis, transdifferentiation of hepatic stellate cells (HSCs), the predominant contributor to fibrogenic hepatic myofibroblast responsible for extracellular matrix (ECM) deposition, is characterized with transcriptional and epigenetic remodeling. We aimed to characterize the roles of H3K27 methyltransferase EZH2 and demethylase JMJD3 and identify their effective pathways and novel target genes in HSCs activation and liver fibrosis.

**Methods:** In primary HSCs, we analyzed effects of pharmacological inhibitions and genetic manipulations of EZH2 and JMJD3 on HSCs activation. In HSCs cell lines, we evaluated effects of EZH2 inhibition by DZNep on proliferation, cell cycling, senescence and apoptosis. In CCl_4_ and BDL murine models of liver fibrosis, we assessed *in vivo* effects of DZNep administration and *Ezh2* silencing. We profiled rat primary HSCs transcriptomes with RNA-seq, screened the pathways and genes associated with DZNep treatment, analyzed EZH2 and JMJD3 regulation towards target genes by ChIP-qPCR.

**Results:** EZH2 inhibition by DZNep resulted in retarded growth, lowered cell viability, cell cycle arrest in S and G2 phases, strengthened senescence, and enhanced apoptosis of HSCs, decreased hepatic collagen deposition and rescued the elevated serum ALT and AST activities of diseased mice, and downregulated cellular and hepatic expressions of H3K27me3, EZH2, α-SMA and COL1A. *Ezh2* silencing by RNA interference *in vitro* and* in vivo* showed similar effects. JMJD3 inhibition by GSK-J4 and overexpression of wild-type but not mutant *Jmjd3* enhanced or repressed HSCs activation respectively. EZH2 inhibition by DZNep transcriptionally inactivated TGF-β1 pathway, cell cycle pathways and vast ECM components in primary HSCs. EZH2 inhibition decreased H3K27me3 recruitment at target genes encoding TGF-β1 pseudoreceptor BAMBI, anti-inflammatory cytokine IL10 and cell cycle regulators CDKN1A, GADD45A and GADD45B, and increased their expressions, while *Jmjd3* overexpression manifested alike effects.

**Conclusions:** EZH2 and JMJD3 antagonistically modulate HSCs activation. The therapeutic effects of DZNep as epigenetic drug in liver fibrosis are associated with the regulation of EZH2 towards direct target genes encoding TGF-β1 pseudoreceptor BAMBI, anti-inflammatory cytokine IL10 and cell cycle regulators CDKN1A, GADD45A and GADD45B, which are also regulated by JMJD3. Our present study provides new mechanistic insight into the epigenetic modulation of EZH2 and JMJD3 in HSCs biology and hepatic fibrogenesis.

## Introduction

Hepatic fibrosis is the common feature of most chronic liver disease and its progression toward cirrhosis is the major cause of liver-related morbidity and mortality. A central hallmark of liver fibrosis is the uncontrolled activation of hepatic stellate cells (HSCs), the predominant contributor to fibrogenic hepatic myofibroblast during liver injury 1-3. HSCs transdifferentiation is associated with dramatic morphological and biological changes including lipid droplets disappearing, enhanced proliferation, excessive collagen deposition and extracellular matrix (ECM) accumulation. Transforming growth factor β 1 (TGF-β1) is the most potent profibrogenic cytokine that promotes HSCs transdifferentiation through activating the canonical TGF-β/SMAD signaling pathway. Activated HSCs could undergo proliferation, senescence, apoptosis and reversion of transdifferentiation 4. Dysregulation and dysfunction of the underlying pathways and genes for phenotypic transition and fate-decision of HSCs are involved in liver fibrogenesis, which could be at least partly ascribed to their epigenetic alterations including DNA methylation, histone acetylation and methylation, microRNAs and long non-coding RNAs 5, 6.

Methylations of histone H3 on lysine 4, lysine 9 and lysine 27 are dynamic and important epigenetic modifications during HSCs activation. Trimethylated histone H3 on lysine 4 (H3K4me3) correlates with genes activation, while H3K9me2/3 and H3K27me2/3 usually lead to gene repression 7. Methyl-CpG binding protein 2 (MeCP2) promotes H3K9 methylation and recruits transcriptional repressor at *PPARγ* gene, a pivotal negative regulator for HSCs transdifferentiation 8, 9. H3K27me3 is executed by polycomb repressive complex (PRC) including enhancer of zeste 2 (EZH2) and suppressor of zeste 12 (SUZ12) as core components. EZH2 is upregulated in activated HSCs, its genetic and pharmacological disruptions phenotypically reduce fibrogenic characteristics of myofibroblasts and attenuate liver fibrogenesis 8. EZH2 inhibition by 3-Deazaneplanocin A (DZNep) has been proposed a proof-of-concept for epigenetic therapy of liver fibrosis, which is one of the most potent *S*-adenosylhomocysteine (AdoHcy) hydrolase inhibitors with broad effects on histone lysine methyltransferase activities, and a widely used H3K27me3 methyltransferase inhibitor of EZH2 for anti-cancer therapeutic development 10. EZH2 is also overexpressed in renal fibrosis 11-13, idiopathic pulmonary fibrosis (IPF) 14, 15, and systemic sclerosis (SSc) 16, a prototypical idiopathic fibrotic disease, its inhibition by DZNep attenuates these fibrotic conditions. GSK126, a highly selective, *S*-adenosyl-methionine-competitive, small-molecule inhibitor of EZH2 methyltransferase activity, was also reported to show therapeutic effects in experimental and clinical settings including lymphoma with EZH2-activating mutations 17 and atrial fibrosis 18. Mechanistically, some target genes of EZH2 and their functions in liver fibrosis has been characterized. For example, PPARγ silencing in HSCs activation is partly ascribed to strengthened H3K27me3 at *PPARγ* chromatin mediated by EZH2 8, 19. However, the functional roles and other novel effective target genes of EZH2 remain to be further elucidated.

Histone methylation is dynamically regulated by methyltransferase and demethylase. We previously reported that the jumonji domain containing protein 1A (JMJD1A), also known as lysine demethylase 3A (KDM3A), modulates HSCs activation and liver fibrosis through demethylating H3K9me2 at *PPARγ* promoter and positively regulating its expression 20. JMJD2D, or KDM4D, was recently reported as a remarkable overexpressed H3K9me2/3 demethylase during HSCs activation, it epigenetically facilitates TLR4 gene transcription and thus activates TLR4/NF-kB signaling pathways, promotes HSCs activation and hepatic fibrosis progression 21. Removal of H3K27me2/3 methylation marks is specifically mediated by the JmjC domain-containing histone demethylases JMJD3 (KDM6B) and UTX (Ubiquitously transcribed Tetratricopeptide repeat on chromosome X) (KDM6A) 22. JMJD3 is involved in cellular processes including differentiation, proliferation, senescence, and apoptosis, and thus is implicated in development and diseases 23. JMJD3, UTX and EZH2 regulate hepatic plasticity inducing retro-differentiation and proliferation of hepatocytes 24. In the setting of SSc, JMJD3, but not UTX, is overexpressed in SSc and murine models of skin fibrosis as well as in cultured fibroblasts, modulates fibroblast activation by regulating H2K27me3 content at *FRA2* gene promoter, which encodes the AP1 transcription factor that regulates ECM production. JMJD3 inhibition by GSK-J4, a specific inhibitor of H3K27me3 demethylase 25, inhibits fibroblast activation and ameliorates experimental fibrosis 26. A recent report also demonstrated that JMJD3 plays direct roles in the pro-fibrotic cardiac fibroblast phenotype and its selective inhibition attenuates cardiac fibrosis 27. But the biological significance and pathological relevance of JMJD3 in HSCs and liver fibrosis is yet unknown.

In the present study, we found that EZH2 and JMJD3 coordinately regulate HSCs activation, pharmacological and genetic abrogation of EZH2 ameliorates liver fibrosis in murine models. Using RNA-seq, we profiled the differential transcriptomes of primary HSCs associated with EZH2 inhibition, and mechanistically identified critical genes involved in liver fibrosis as novel direct targets under the epigenetic regulations of EZH2 and JMJD3, including the TGF-β1 pseudoreceptor BAMBI gene.

## Results

### EZH2 and JMJD3 regulate HSCs activation

**Figure 1:**
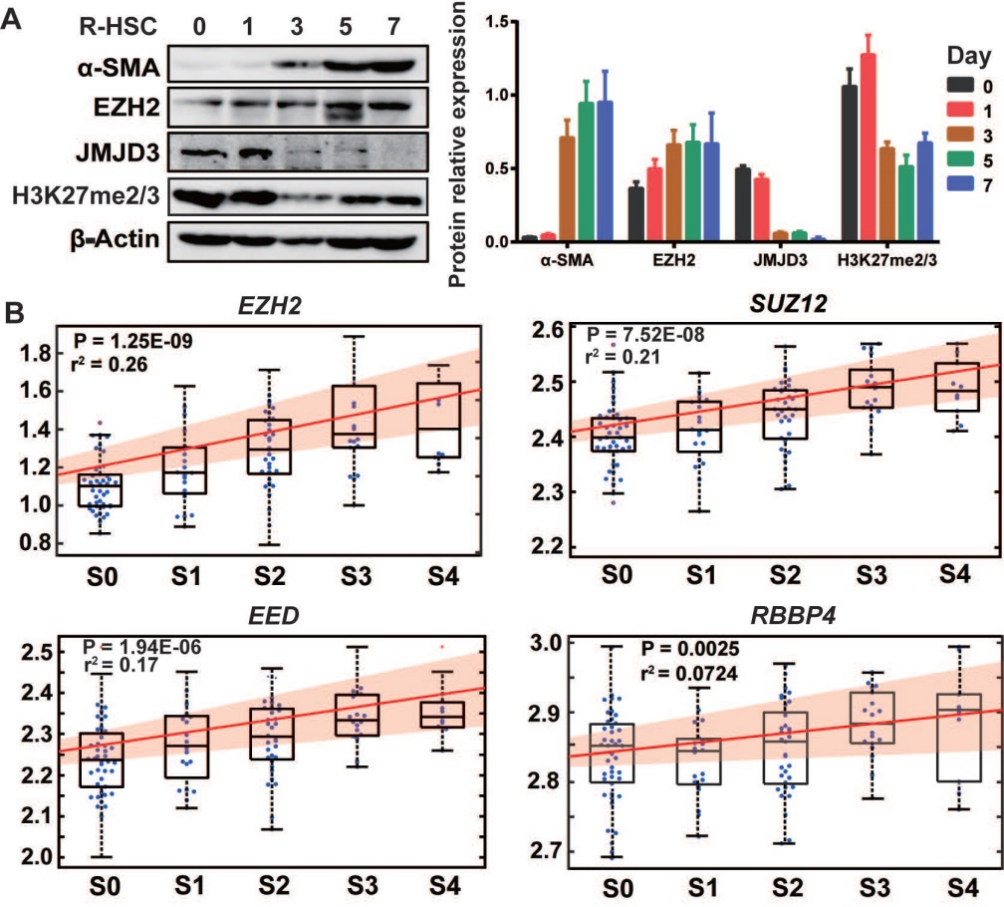
The association of EZH2 and JMJD3 expressions with HSC activation and liver fibrosis. (**A**) The expressions of EZH2, JMJD3, α-SMA, H3K27me2/3 and β-Actin as inner control during culture-induced activation of rat primary HSCs (0, 1, 3, 5, 7 days) was analyzed by Western blot. (**B**) In the gene arrays dataset for liver biopsy samples from 124 liver fibrosis patients (GSE84044, ref 29), linear regression was used to analyze the association of hepatic expression of PRC2 subunit genes (*EZH2*, *SUZ12*, *EED* and *RBBP4*) with sequential histological staging of fibrosis, Scheuer score, *S*, which is divided as S0 (stage 0, *n* = 43), S1 (*n* = 20), S2 (*n* = 33), S3 (*n* = 18) and S4 (*n* = 10). The box plot and fitting curve showed the association trend. The significance of correlation was expressed as* r*-squared values of regression and *P* < 0.01 was considered as statistical significance.

We first isolated rat primary HSCs, monitored culture-induced HSCs activation, and analyzed proteins expressions of EZH2, JMJD3, UTX and H3K27me2/3 (Figure 1A). EZH2 expression moderately increased during HSC activation, while JMJD3 was positively expressed in quiescent HSCs but was rapidly silenced and almost undetectable in activated HSCs. The expression of UTX in primary and activating HSCs was undetectable, suggesting possible redundancy in HSCs biology for the two H3K27me2/3 demethylases. In public transcriptome datasets cross human tissues 28, *UTX* transcript level in liver is relatively low, and a recent study also showed that UTX is deregulated during hepatic differentiation 24. In line with previous finding that H3K27me2 is sharply silenced and H3K27me3 is clearly increased in activated rat primary HSCs as compared with quiescent HSCs 7, we observed modest reduction in the pooled H3K27me2/3 level during HSCs activation. In multiple hepatic cell types including human (LX-2) and mouse (JS1) HSCs, we found ubiquitous and relatively high expression of EZH2 but the expression of JMJD3 was almost undetectable (Figure S1A). It has been also reported that rat *Ezh2* transcription is induced both in cultured myofibroblasts and in HSCs that are transdifferentiating *in vivo* in response to BDL and CCl_4_ injury, while *Jmjd3* transcription is downregulated during HSCs activation 7, 8. In the previously reported hepatic transcriptome expression data for 124 liver fibrosis patients (GSE84044 in NCBI GEO) 29, we found that transcriptions of *EZH2* (*r*^2^ = 0.26, *P* = 1.25E-09) and other PRC2 components genes, *SUZ12*, *EED* and *RBBP4* were all positively correlated with sequential histological staging of fibrosis (Scheuer score, *S*) (Figure 1B), while no significant correlation was observed for *JMJD3* (data not shown). The expression pattern of EZH2 and JMJD3 during HSCs activation and liver fibrosis progression indicates that they might be involved in HSCs biology and have pathological relevance.

**Figure 2:**
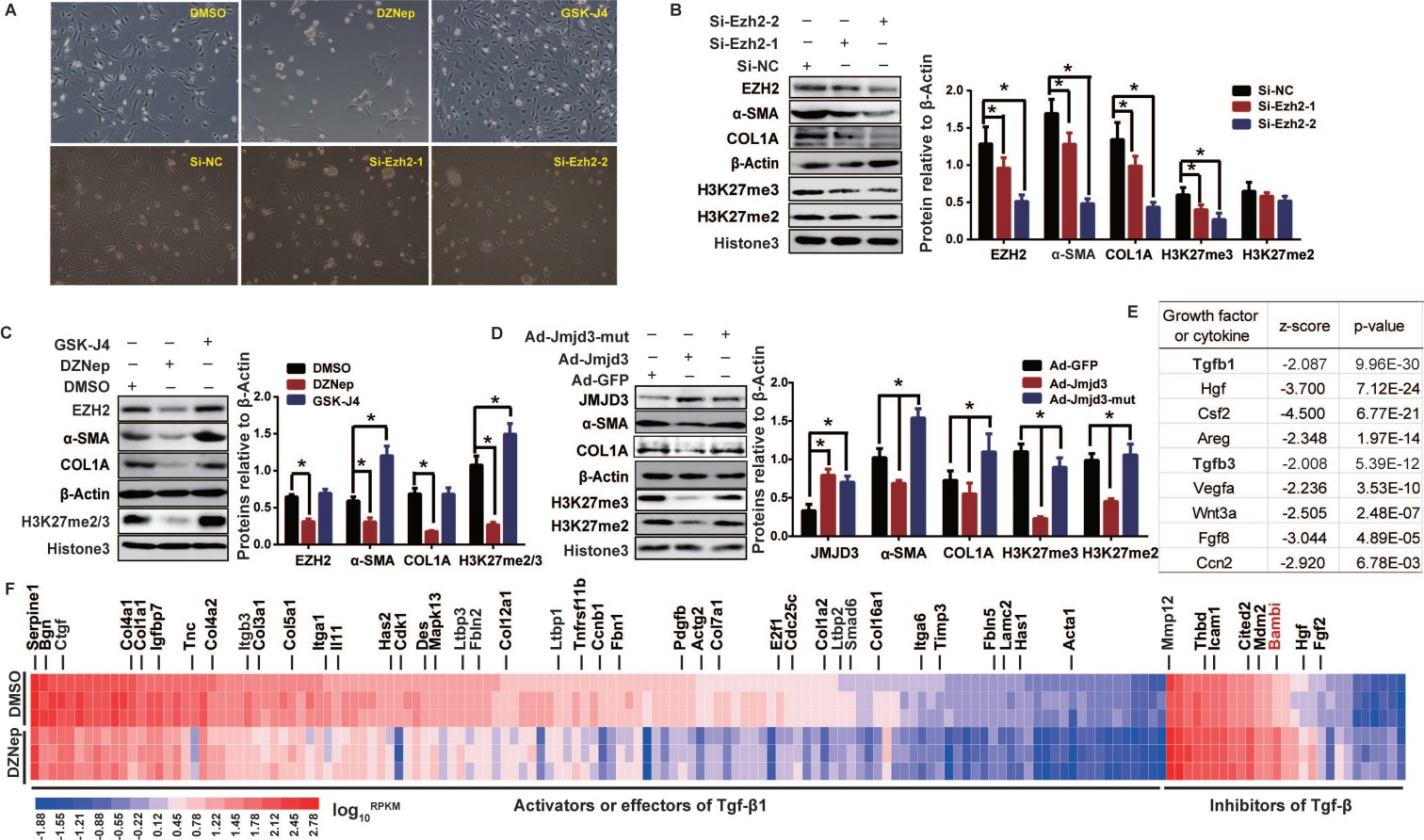
EZH2 and JMJD3 regulate HSC activation through modulating Tgfβ1 signaling pathway. Rat primary HSCs were cultured and treated with inhibitors of EZH2 (DZNep) or JMJD3 (GSK-J4), or transfected with siRNAs of *Ezh2*, or infected with adenovirus recombined with *Jmjd3*. RNA-seq was conducted for HSCs treated with DZNep or DMSO as control. (**A**) HSCs growth and morphology were assessed in fifth day after administration, the magnification is 200×. (**B-D**) EZH2, JMJD3, α-SMA and COL1A expressions and H3K27 methylation level were determined by Western blot with β-ACTIN and Histone 3 as inner controls respectively. * *P* < 0.05. (**E**) Basing on the DEGs associated with DZNep treatment in HSCs, the upstream regulator analysis of IPA identified Tgfβ1 among the most significantly inhibited growth factors or cytokines. The top candidates are shown with overlap *P* value < 0.01 and | activation *Z*-score | >2. (F) The heatmap shows transcriptional value log_10_*^RPKM^* for genes encoding effectors, activators and inhibitors of TGFβ1 signaling pathway. *Bambi* in the inhibitor panel was highlighted in red.

We therefore evaluated, in rat primary HSCs and mouse JS1 cell line, the effects of pharmacological inhibition and genetic manipulation of EZH2 and JMJD3 on morphological and biochemical phenotypes of HSCs activation. Treatment of primary HSCs with DZNep, or transient transfection with potent siRNAs for *Ezh2* silencing, resulted in more quiescent HSCs-phenotype and pronounced growth retardation (Figure 2A), remarkably weakened H3K27me2/3, and significantly downregulated α-SMA and COL1A (Figure 2B-C). These epigenetic and biochemical changes were also observed in primary HSCs with adenovirus-mediated stable overexpression of wild-type *Jmjd3*, but not of its demethylation-defective mutant (Figure 2D). Similarly, *Ezh2* silencing in JS1 cells with retrovirally-expressed shRNAs significantly weakened H3K27me2/3 and downregulated α-SMA and COL1A, while stable overexpression of wild-type *Ezh2*, but not of its methylation-defective mutant, significantly upregulated α-SMA and COL1A (Figure S1B-C). In contrast, treatment of primary HSCs with GSK-J4, an jumonji H3K27me3 demethylase inhibitor of JMJD3 30, resulted in enhanced cell growth, morphological transition from quiescent HSCs to myofibroblast, reinforced H3K27me3, and upregulated fibrotic markers (Figure 2A, C). These data suggest that EZH2 and JMJD3 regulate HSCs activation.

**Figure 3:**
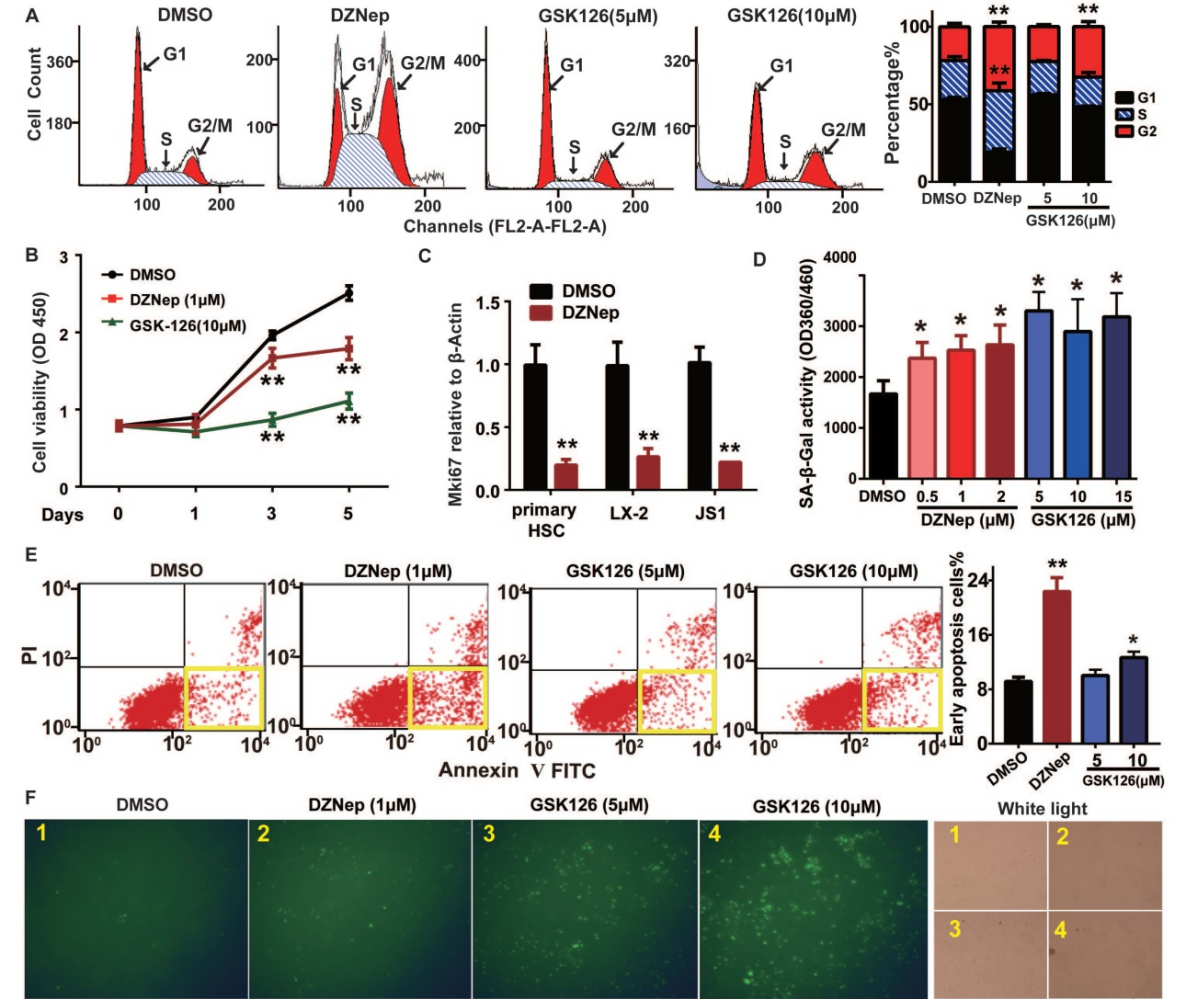
Cellular phenotypic effects of EZH2 inhibition with DZNep or GSK126 on HSCs cell cycling, proliferation, senescence and apoptosis. JS1 cells were administrated with DZNep, GSK126 or DMSO and subjected to analyses of cell cycle (**A**), cell growth (**B-C**), senescence (**D**) and apoptosis (**E-F**) with staining and flow cytometry. The transcriptional expression of cell proliferation marker MKI67 gene in rat primary HSC, human LX-2 and mouse JS cells were measured by RT-qPCR (C). Cell senescence was determined by measuring SA-β-Gal activity (D). The apoptotic cells were determined by Annexin-V-PI double staining (E) and FITC-dUTP staining by TUNEL apoptosis assay (F).The yellow frames in panel E present early apoptotic cells. Data were measured from triple independent experiments. * *P* < 0.05, ** *P* < 0.01.

Because EZH2 is expressed in activated HSCs and its hepatic expression positively correlates with fibrosis staging, we thus focused on the phenotypic relevance of pharmacologic and genetic abrogation of EZH2 *in vitro* and *in vivo*. EZH2 inhibition by DZNep in mouse JS1 cells resulted in cell cycle arrest in S and G2 phases (Figure 3A), lowered cell viability (Figure 3B), increased cell senescence (in dose dependent mode) (Figure 3D), and higher percent of early apoptotic cells (Figure 3E-F). Similar effects on these cellular phenotypes of DZNep were also observed in human LX-2 cells (Figure S1D-F). Of note, DZNep treatment in rat primary HSCs, JS1 and LX-2 cells significantly lowered the transcriptional expression of cell proliferation marker protein Ki-67 coding gene MKI67 (marker of proliferation Ki-67) (Figure 3C). On the other hand, treatment of JS1 cells with GSK126 also increased cell senescence but not in dose dependent fashion (Figure 3D). High dose (10 μM) GSK126 treatment in both JS1 (Figure 3A, E-F) and LX-2 (Figure S1E-F) significantly increased the percentages of the cells in G1 phase and of early apoptosis cells. However, high dose (10 μM) GSK126 treatment sharply reduced the cell viabilities of JS1 (Figure 3B) and LX-2 (Figure S1D), suggesting possible cytotoxicity of GSK126 towards HSCs. These results suggest that EZH2 is one key epigenetic regulator for HSCs proliferation, senescence and apoptosis, its inhibition with DZNep could modulate these cellular phenotypes.

In order to uncover the underlying pathways and genes for broader effects of DZNep on HSCs phenotypes, we profiled the transcriptomes of rat primary HSCs treated with DZNep using RNA-seq. We identified 2,639 (806 upregulated and 1,833 downregulated) associated DEGs, the transcriptions of 43 candidates among them were also validated with RT-qPCR (Figure S2). Given that quiescent HSC is activated by extracellular mediators including cytokines and growth factors that trigger intracellular downstream effectors 4, we used IPA URA to investigate the effect of DZNep treatment on functionality of upstream regulators. For the observed DEGs, IPA identified Tgfβ1 and Tgfβ3, two well-defined pivotal profibrogenic cytokines, among the most significantly inhibited upstream regulators with molecule type as growth factor or cytokine (Bonferroni corrected *P* < 0.01). 154 out of the 351 associated DEGs in knowledgebase have measurement direction consistent with Tgfβ1 inhibition (*P* = 9.96E-30, *Z*-score = -2.087), the majority (127) of which are downregulated activator or effector genes, while a few (27) are upregulated inhibitor genes (Figure 2E-F). Because EZH2 inhibition could supposedly release the repression of its direct target genes, we therefore emphasized on the upregulated inhibitor genes of Tgfβ1, and found that DZNep treatment resulted in significant upregulation of *Bambi* (bone morphogenetic protein and activin membrane bound inhibitor), the TGF-β1 pseudoreceptor gene and the key negative regulator of TGFβ signaling 31. These data suggest that EZH2 inhibition by DZNep in HSCs could pervasively attenuate TGF-β/SMAD signaling pathway through, in part at least, modulating the expression of its regulators such BAMBI.

**Figure 4:**
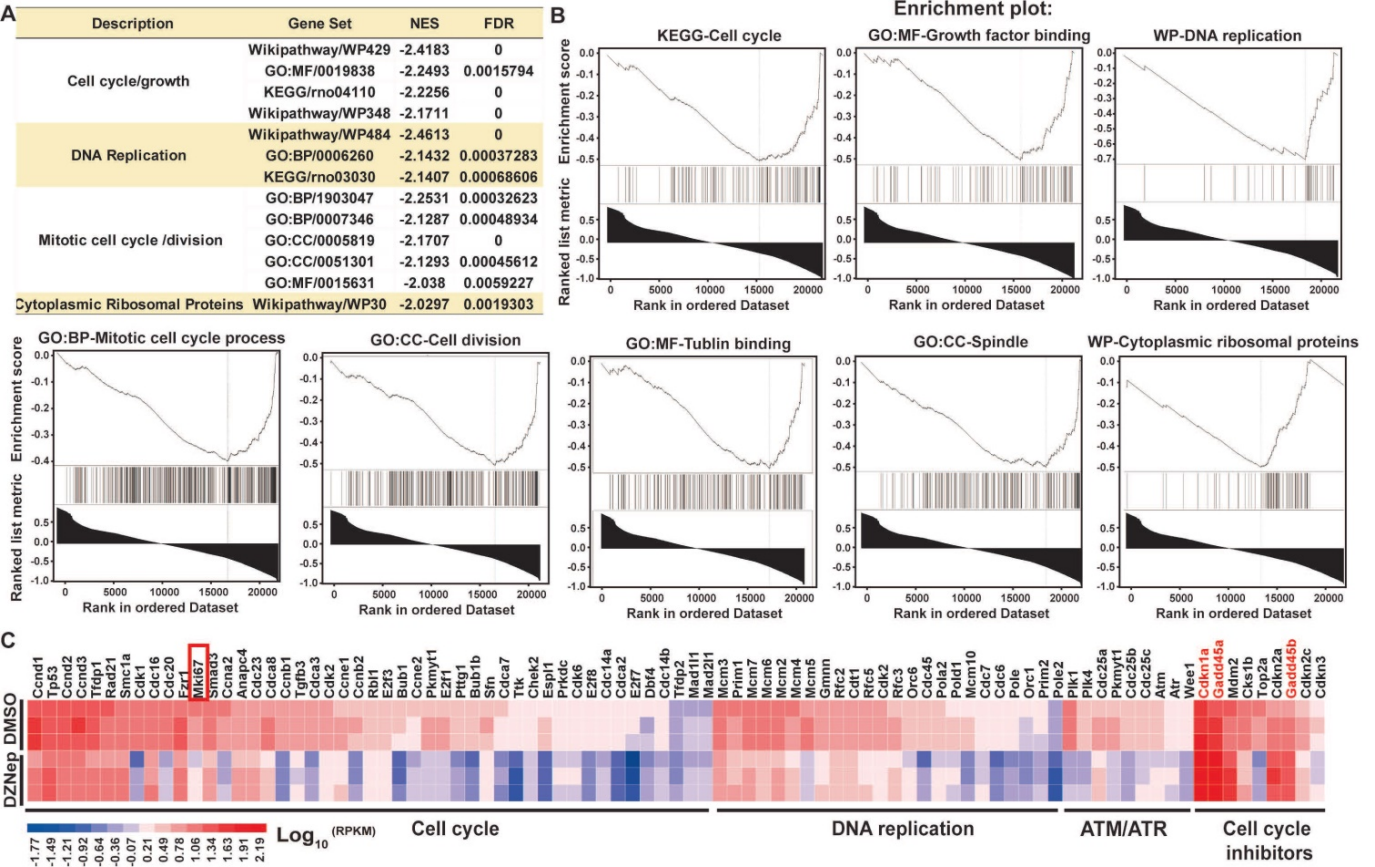
DZNep treatment of HSCs disturbs the transcriptional expressions of cell cycle regulator genes. Rat primary HSCs were treated with DZNep or DMSO, their transcriptomes were compared with RNA-seq profiling. The pre-ranked gene list was subjected to bioinformatics analysis tool GSEA. Significant enrichment of gene sets related to regulation of cell cycle, DNA replication, cell division and ribosomal proteins were demonstrated (**A-B**). NES: normalized enrichment score, FDR: false discovery rate. (C) The heatmap shows expression values of genes involved in cell cycle regulation. *Mki67* as one well-defined cell proliferation marker in the cell cycle panel, *Cdkn1a*, *Gadd45a* and *Gadd45b* in the cell cycle inhibitor panel were highlighted.

To ascertain the thematic association of gene signature for DZNep treatment, we conducted functional enrichment analysis. Interestingly, key signaling pathways involved in cell cycle, DNA replication, mitotic cell cycle and spindle organization, and cytoplasmic ribosomal proteins were significantly inactivated (Figure 4A-B). Specifically, some positive regulators of cell cycle including cyclins, cyclin-dependent kinases and E2F transcription factors, and of mitosis were pervasively downregulated; while the key negative regulators of cell cycle, *Cdkn1a* (cyclin dependent kinase inhibitor 1A), *Gadd45a* (growth arrest and DNA damage inducible alpha) and *Gadd45b*, the target genes of p53 and primary inhibitors of G2/M transition) were upregulated (Figure 4C). These results indicate that DZNep-induced cycle arrest, proliferation inhibition and apoptosis of HSCs might be ascribed to the perturbed expression of cell cycle regulators such as CDKN1A and GADD45.

### Pharmacological and genetic abrogation of EZH2 ameliorates liver fibrosis *in vivo*

**Figure 5:**
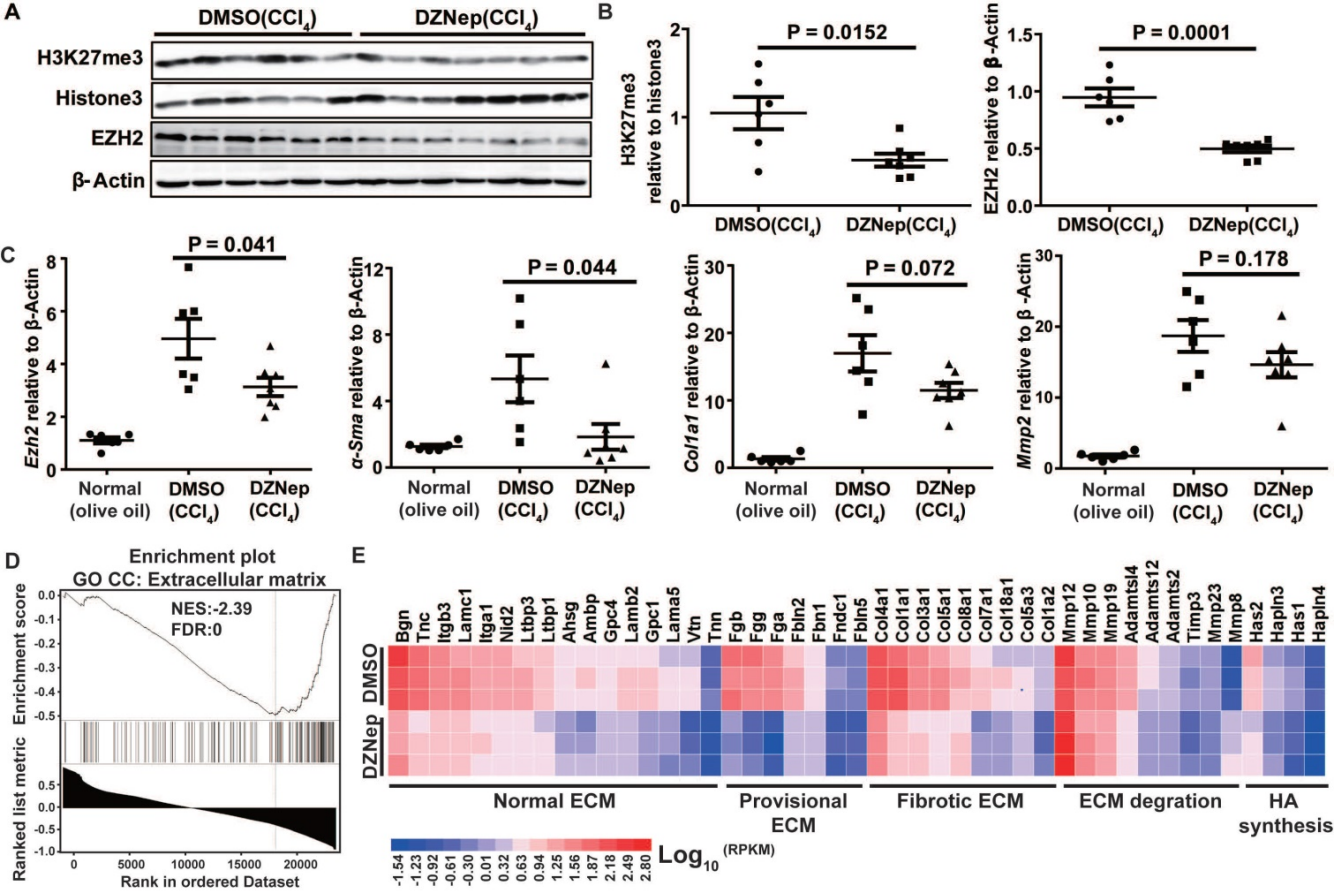
DZNep treatment results in reduced hepatic expressions of fibrotic marker genes in mice with CCl_4_-induced liver fibrosis and prevalent downregulation of ECM component genes in HSCs. Hepatic expressions of H3K27me3, Histone3, EZH2 and β-Actin in CCl_4_ mice with DZNep or DMSO treatment were determined by Western blot (**A-B**). Hepatic transcriptions of *Ezh2*, *α-Sma*, *Col1a*, *Mmp2* and *β-Actin* were determined by RT-qPCR. The pre-ranked gene list for transcriptomic profiling of rat primary HSCs treated with DZNep or DMSO was subjected to GSEA, ECM genes were significantly enriched (**C**). The heatmap shows transcriptional changes of various ECM component genes (**D**). Statistical significance was determined with Student's* t*-test in independent samples.

**Figure 6:**
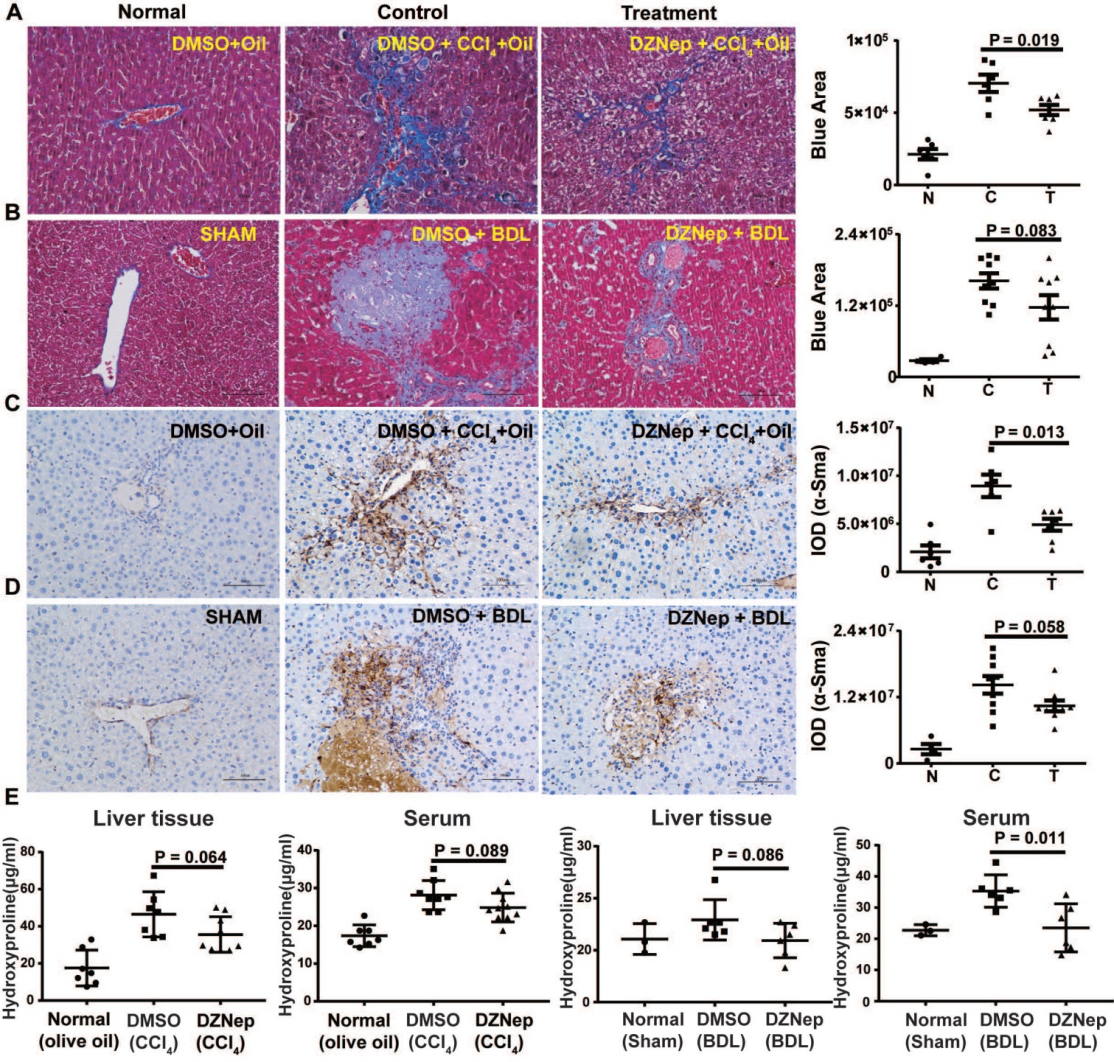
DZNep treatment ameliorates hepatic fibrogenesis in mice. The effect of DZNep on hepatic fibrosis induced by CCl_4_ (**A**) or BDL (**B**) was measured by Masson's trichrome staining. The effect of DZNep on HSCs activation *in vivo* was measured by immunohistochemistry of α-SMA (**C-D**). The effect of DZNep on the collagen level was measured by concentration of hydroxyproline in liver tissues and serum (**E**).

**Figure 7:**
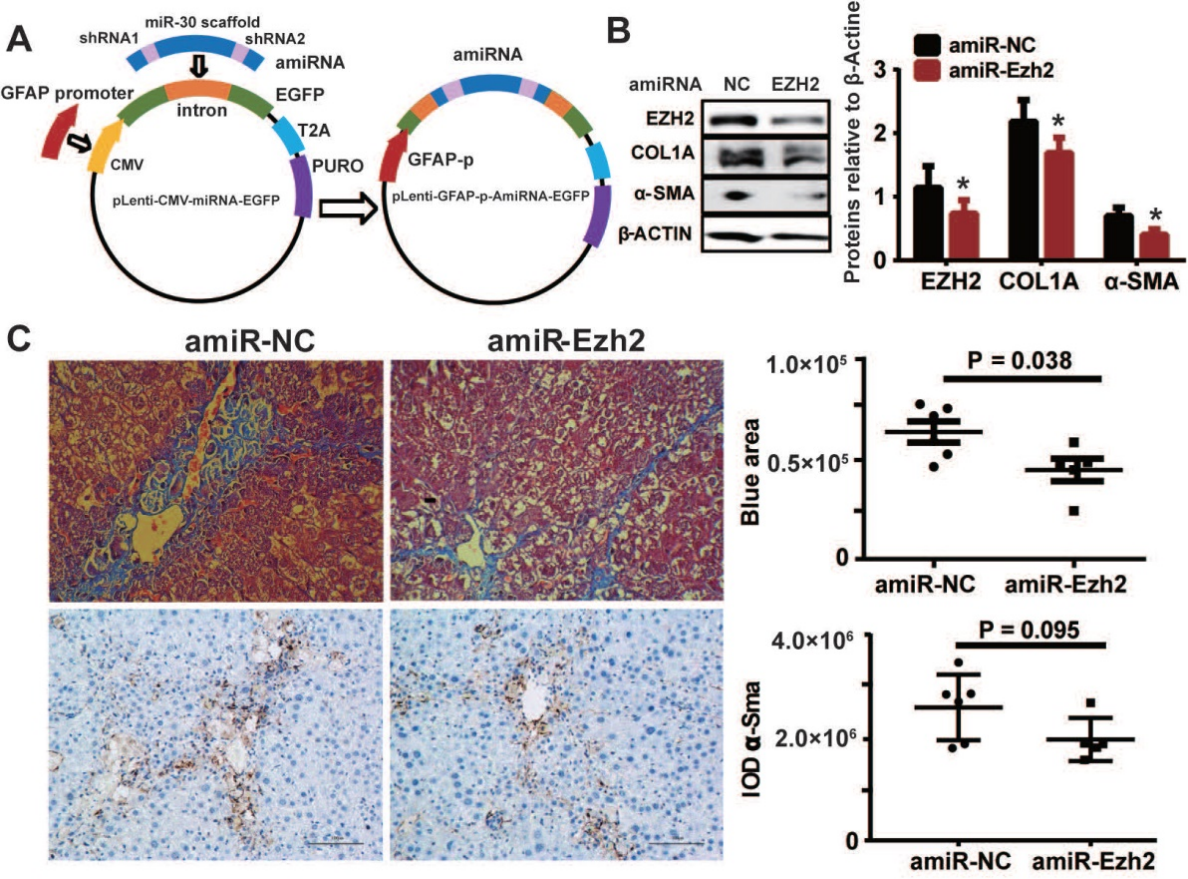
HSC-specific *Ezh2* silencing *in vivo* ameliorated liver fibrosis. (**A**) Construction of amiRNA vector for *Ezh2* silencing. The amiRNA consisted of miR-30a backbone as structural scaffold and two concatenated pre-miR30-shRNA cassettes embedding valid interfering shRNA (amiR-*Ezh2*) or negative control shRNA (amiR-NC) as cargos. Based on lentiviral microRNA expressional vector, amiRNA was inserted into the cloning site located in *EGFP* intron, the original CMV promoter was substituted with human *GFAP* promoter. (**B**) *Ezh2* silencing in JS1 cells was achieved by infection of lentiviral-amiR-*Ezh2*, expressions of EZH2, COL1A, α-SMA and β-ACTIN were determined by Western blot. (**C**) In CCl_4_ mice, lentivirally expressed amiRNA was transduced into the liver through tail-vein injection, therapeutic effects of amiRNA-based HSC-specific *Ezh2* silencing was analyzed by measuring collagens deposition with Masson's trichrome staining and α-SMA expression with immunohistochemistry.

To decipher the pathological relevance of EZH2 in hepatic fibrosis, we investigated *in vivo* phenotypes of pharmacological inhibition and genetic knockdown of EZH2 in two murine models of liver fibrosis (CCl_4_ and BDL). DZNep administration in diseased mice with CCl_4_ significantly downregulated the expressions of EZH2 (mRNA, *P* = 0.041; protein, *P* = 0.0001) and H3K27me3 (*P* = 0.015) in liver (Figure 5A-C). We observed consistently downregulated hepatic transcriptional expression of fibrotic marker genes *α-Sma*, *Col1a1* and *Mmp2* in treatment mice (Figure 5C). The Masson staining results showed that collagen deposition was significantly reduced in DZNep-treatment panels of both CCl_4_ (*P* = 0.019) and BDL (*P* = 0.083) mice (Figure 6A-B). Consistently, the IOD of positive α-SMA areas, the marker of *in-situ* activated HSCs, was also significantly decreased in treatment panel (*P* = 0.013 and 0.058 for CCl_4_ and BDL models respectively) (Figure 6C-D). DZNep treatment also decrease the content of hydroxyproline in liver tissues and serum both in CCl_4_ (*P* = 0.064 in liver tissues and* P* = 0.089 in serum) and BDL models (*P* values being 0.086 and 0.011 respectively) (Figure 6E). Furthermore, we also investigated the effect of *Ezh2* silencing *in vivo* on liver fibrogenesis. Based on our recently proposed amiRNA strategy 32, we designed the lentivirally expressed and HSC specific *GFAP* gene promoter driven amiRNA system (amiR-*Ezh2*) for targeted *Ezh2* silencing (Figure 7A). The JS1 cells infected with this lentiviral-amiR-*Ezh2* showed efficient *Ezh2* silencing and significant downregulation of COL1A and α-SMA (Figure 7B). In CCl_4_ mice, hepatic delivery and transduction of recombinant lentivirus particles through tail vein injection, resulted in pronounced reductions in collagens deposition (*P* = 0.038) and α-SMA expression* in-situ* (*P* = 0.095) in fibrotic tissue (Figure 7C). Consistent with the therapeutic effect of DZNep *in vivo*, in the transcriptomic analysis of rat primary HSCs treated with DZNep, we found striking inactivation of ECM related gene set for the GO category “extracellular matrix” by GSEA (Figure 5D). A variety of ECM components genes at various fibrotic stage were globally downregulated, including normal ECM components, provisional ECM components, fibrotic ECM components, and the synthase genes for hyaluronan, a ubiquitous component of different stages of ECM considered as a biomarker and driving factor for fibrosis 33 (Figure 5E). Collectively, the *in vitro* and *in vivo* data suggest that EZH2 inhibition by DZNep attenuates TGF-β/SMAD signaling in HSCs, inhibits HSCs activation, mitigates hepatic ECM accumulation and eventually ameliorates liver fibrosis.

### EZH2 inhibition by DZNep alleviates liver injury and epigenetically up-regulates *Il10*

**Figure 8:**
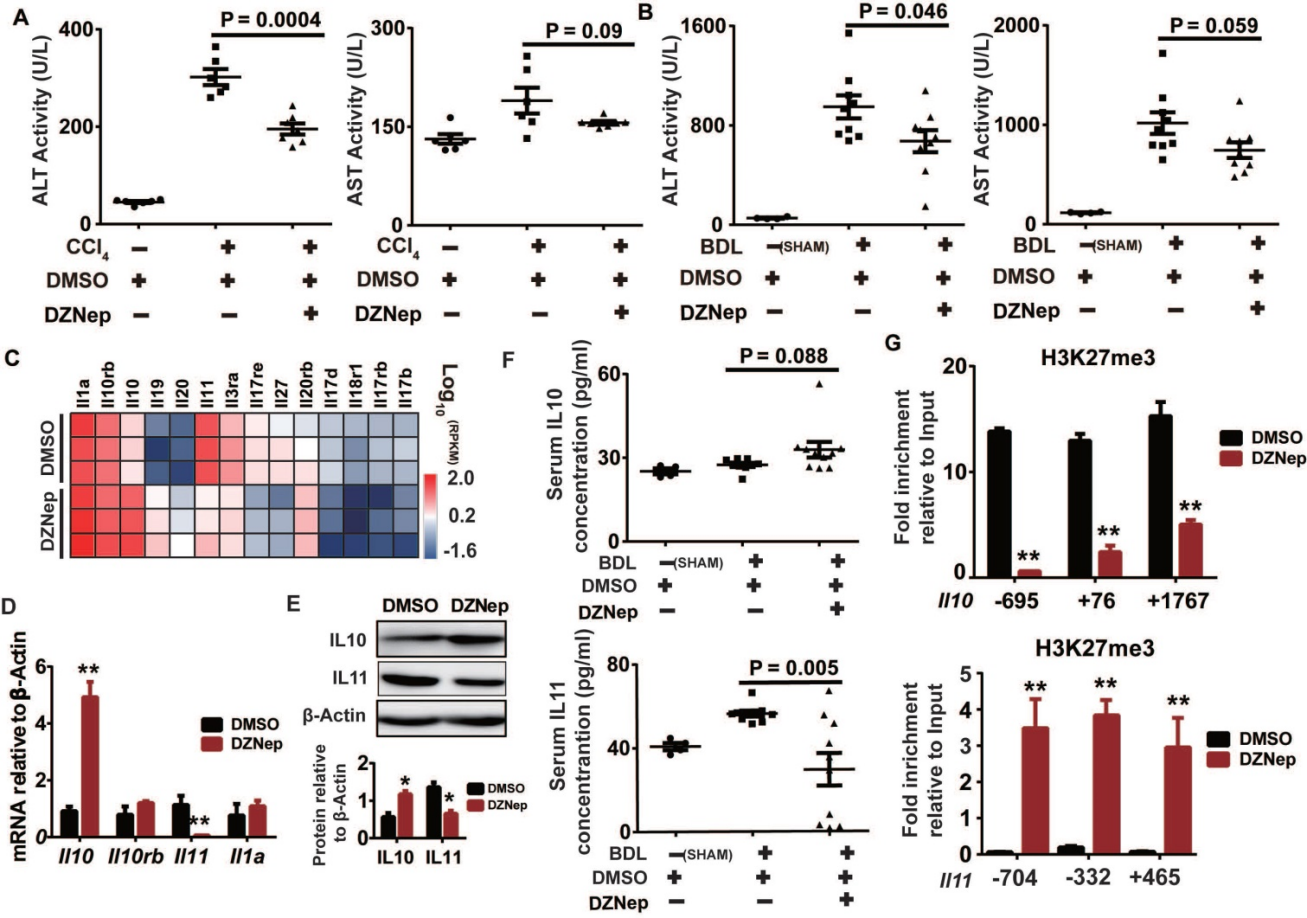
DZNep treatment confers protection against liver injury and correlates with IL10 upregulation and IL11 downregulation. Serum ALT and AST activities of CCl_4_ (**A**) and BDL mice (**B**) treated with DZNep or DMSO was measured by biochemistry analyzer. The heatmap shows transcription changes of interleukin related genes in rat primary HSCs associated with DZNep treatment (**C**), among which transcription expressions of *Il10*, *Il11*, *Il1a*, and *Il10rb* were validated by RT-qPCR (**D**), and protein expressions of IL10 and IL11 were measured by Western blot (**E**). Serum concentration of IL10 and IL11 of BDL mice and controls were measured by ELISA (**F**). Enrichments of H3K27me3 at gene bodies of *Il10* and *Il11* in rat primary HSCs treated with DZNep or DMSO were determined by ChIP-qPCR (**G**). Statistical significance was determined by Student's *t*-test. * *P* < 0.05, ** *P* < 0.01.

HSC is not only passive target of pro-fibrogenic factors but also central modulator of hepatic inflammation by secreting cytokines, which is profoundly linked to liver injury and fibrosis 34. In the above *in vivo* study, DZNep administration significantly ameliorated the experimentally-induced elevation in activities of ALT (*P* = 0.0004 and 0.046) and AST (*P* = 0.09 and 0.059) in both CCl_4_ and BDL mice, which serve as surrogates of hepatocellular damage (Figure 8A-B). Consistently, DZNep treatment of rat primary HSCs was associated with overall downregulation of *Il17b*, *Il17rb*, *Il17d* and *Il17re* genes (Figure 8C), which code components of IL17 signaling that not only directly induces collagen type I production in HSCs, but also is involved in promoting liver damage and fibrosis resulting from inflammasome activation 35, 36. Particularly notable, DZNep treatment of HSCs resulted in significant upregulation of *Il10*, a well-defined anti-inflammatory cytokine 37-39, and remarkable downregulation of *Il11*, a putative pro-fibrogenic cytokine 40. In accordance, DZNep treatment of BDL mice correlated with elevated and declined serum concentrations of IL10 (*P* = 0.088) and IL11 (*P* = 0.005) respectively (Figure 8F). Mechanistically, DZNep treatment in rat primary HSCs remarkably decreased H3K27me3 enrichment at *Il10* gene body (Figure 8G), and thereby significantly increased its transcript and protein expressions (Figure 8D-E). However, although consistent with the significant reduction in the mRNA and protein expression of IL11 gene (Figure 8D-E), the sharply strengthened occupancy of H3K27me3 at *Il11* gene body (Figure 8G) associated with DZNep treatment, is apparently contrary to the inhibitory effect of DZNep towards the H3K27 methyltransferase activity of EZH2. This discrepancy could be possibly ascribed to the broad effects of DZNep on histone lysine methyltransferase activities, suggests the complex H3K27me3 homeostasis and epigenetic regulation on IL11 gene expression. These data suggest that EZH2 inhibition by DZNep alleviates experimental liver injury partial through epigenetically regulating *Il10*.

### EZH2 and JMJD3 regulate *Bambi*, *Cdkn1a* and *Gadd45* genes in HSCs

**Figure 9:**
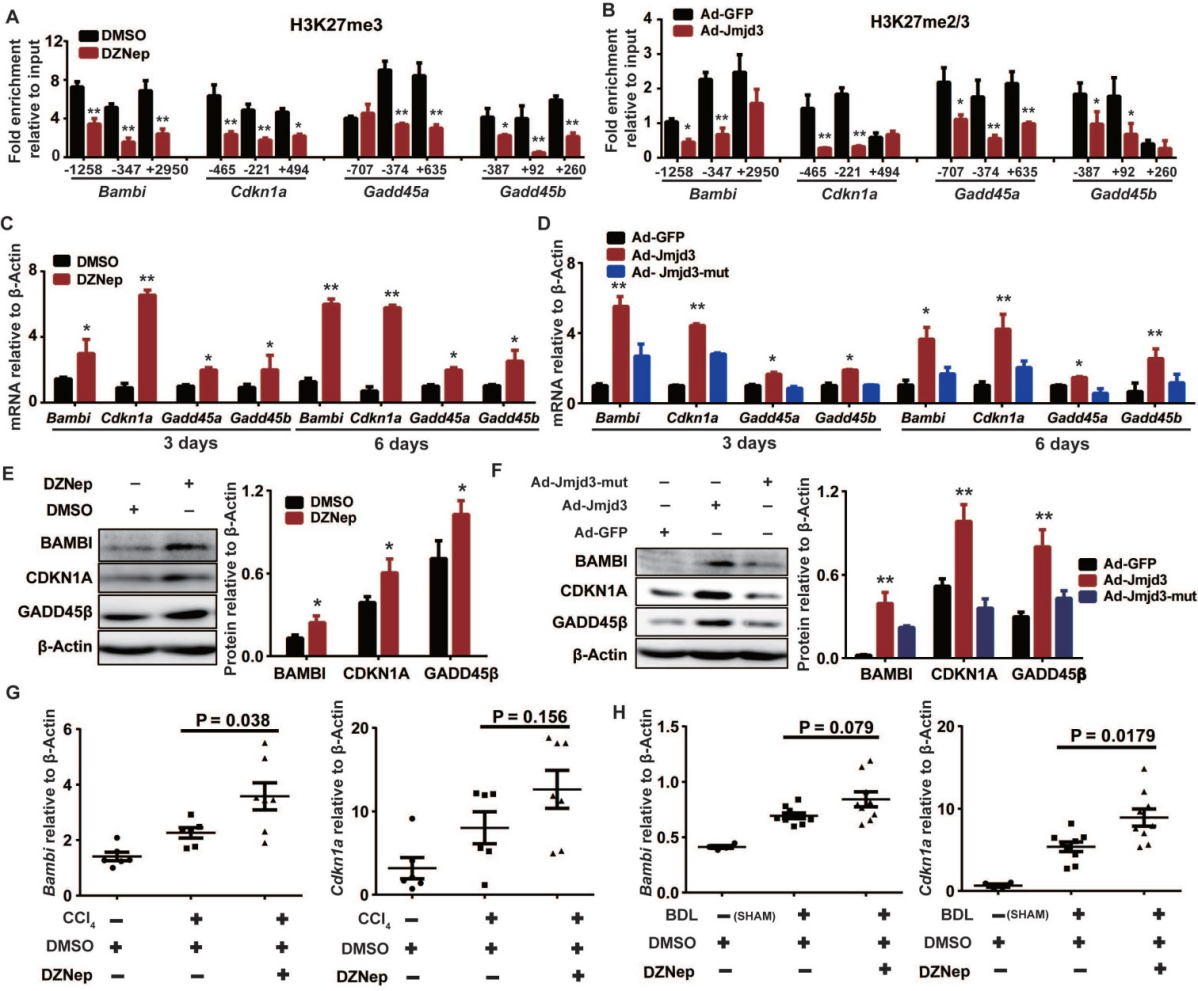
EZH2 and JMJD3 regulate *Bambi*, *Cdkn1a*, *Gadd45a* and *Gadd45b.* Primary rat HSCs were treated with DZNep or infected with adenovirus-*Jmjd*3 or their controls respectively. The effects of DZNep on H3K27me3 (**A**) or *Jmjd3* overexpression on H3K27me2/me3 (**B**) enrichments at target genes were determined by ChIP-qPCR. The 5′ endpoints of PCR products are positioned as upstream (-) or downstream (+) from transcription start sites. The effect of DZNep treatment or *Jmjd3* overexpression on transcript and protein products of target genes were determined by RT-qPCR (**C-D**) and Western blot (**E-F**) respectively. β-Actin was used as internal control. Hepatic transcriptions of *Bambi* and *Cdkn1a* in CCl_4_ (**G**) and BDL (**H**) mice were determined by RT-qPCR. * *P* < 0.05, ** *P* < 0.01.

Given that H3K27m3 is usually silencing epigenetic mark that could be coordinately regulated by EZH2 and JMJD3, we hypothesized that some upregulated DEGs associated with DZNep treatment of HSCs might be common targets of EZH2 and JMJD3, which could be exemplified by *Bambi*, *Cdkn1a*, *Gadd45a* and *Gadd45b* that are involved in regulation of HSCs activation and proliferation. Indeed, DZNep-mediated EZH2 inhibition and adenovirus-mediated *Jmjd3* overexpression in rat primary HSCs both consistently decreased H3K27me3 enrichment at promoters and gene bodies of *Bambi*, *Cdkn1a*, *Gadd45a* and *Gadd45b*, increased their transcriptional expression both at early (3 days) and late (6 days) stage of HSCs activation (Figure 9A-D). We also observed substantial increase in protein expression for BAMBI, CDKN1A and GADD45B in HSCs with DZNep treatment or with overexpression of wild-type but not mutant *Jmjd3* (Figure 9E-F). Furthermore, DZNep treatment in CCl_4_ and BDL mice correlated with consistently upregulated hepatic transcription of *Bambi* (*P* = 0.038 and 0.079) and *Cdkn1a* (*P* = 0.156 and 0.018) (Figure 9G-H). The *in vitro* and *in vivo* data collectively suggest that EZH2 and JMJD3 coordinately modulate HSCs activation and liver fibrosis through, at least in part, epigenetically regulating *Bambi*, *Cdkn1a*, *Gadd45a* and *Gadd45b*.

## Discussion

HSCs activation is the core event of hepatic fibrogenesis. The phenotypic transition of quiescent HSCs (qHSCs) to myofibroblasts is underpinned by transcriptional remodeling, which could be epigenetically regulated by histone modifications. In this study, we characterized divergent expressions and antagonistic functions of histone H3K27 methylase EZH2 and demethylase JMJD3 in HSCs activation and liver fibrosis. We showed that EZH2 and JMJD3 contrastingly regulated HSCs activation, EZH2 inhibition by DZNep or its silencing by siRNA repressed HSCs activation and proliferation, while *Jmjd3* overexpression also achieved these effects. Furthermore, DZNep-treatment or HSC-targeted *Ezh2* silencing by amiRNA in diseased mice ameliorated experimental liver fibrosis. To our best knowledge, this study is among the few that have investigated the roles of both methyltransferase and demethylase for histone epigenetic modification in liver fibrosis.

One of our novel findings is that EZH2 and JMJD3 regulate HSCs activation partially at least through epigenetically modulating the expression of *BAMBI*, the key negative regulator of TGFβ signaling. BAMBI is a TGFβ superfamily type Ⅰ receptor that lacks intracellular kinase domain and therefore blocks the signal transduction 31. BAMBI also forms a ternary complex with SMAD7 and TGFβ type I receptor, impairs SMAD3 activation and thus inhibits signal transduction 41. BAMBI is expressed at high levels in quiescent HSCs but at low levels in *in vivo*-activated HSCs isolated from CCl_4_ and BDL mice; its overexpression prevents HSCs activation, whereas its dominant negative form leads to strongly sensitized HSCs to TGFβ 42. Hepatic *BAMBI* expression in liver fibrosis patients with advanced stage is lower as compared to those with mild or no fibrosis 43. In murine liver fibrosis, intestinal bacterial endotoxin lipopolysaccharide and dietary cholesterol stimulate Toll-like receptor 4, induce occupancy of NF-κBp50 and HDAC1 on *Bambi* promoter, and downregulate its expression in HSCs, leading to enhanced TGFβ signaling and increased HSCs activation and hepatic fibrosis 42, 44, 45. Interestingly, as a key mediator in TGF-β1-driven HSCs activation, EZH2 itself is upregulated in HSCs treated with TGF-β1 46. In fibroblasts from human lung affected with idiopathic pulmonary fibrosis, TGF-β1 treatment significantly increases the association of EZH2 to its target gene and reinforces epigenetic repression 47. It is therefore plausible that DZNep treatment of HSCs could disrupt EZH2-mediated epigenetic repression of *BAMBI*, then strengthen the inhibition of TGF-β1 signaling, and thereby result in reduced EZH2 expression. Indeed, we here and other groups have consistently observed that DZNep not only inhibits EZH2 activity but also decreases its expression in cells including HSCs, SSc dermal fibroblasts, lung fibroblasts, renal fibroblasts, and in fibrotic tissues from diseased animals including liver, lung and kidney 11, 13, 15, 16, 48.

The epigenetic modulation of TGFβ signaling by EZH2 could be evidenced by the observations that DZNep treatment of HSCs resulted in prevalent reduction in ECM expression, whose remodeling and turnover are among the key characteristics of activated HSCs, and pervasive downregulation of its downstream effector genes including *Il11*. *IL11* upregulation is the dominant transcriptional response of primary cardiac fibroblasts to TGFβ1 exposure, fibroblast-specific* Il11* transgene expression or IL11 injection in mice causes heart and kidney fibrosis, whereas its genetic abrogation protects against disease 40. *IL11* is transcriptionally regulated by TGFβ1 and IL1A in pulmonary fibroblasts and is highly upregulated in fibroblasts from patients with systemic sclerosis 49, 50. Interestingly, we here report that *Il11* gene and its upstream regulator *Il1a* gene were highly transcribed in cultured primary HSCs, DZNep-treatment significantly down-regulated *Il11* but did not affect *Il1a* expression (Figure 8C-D). Furthermore, in BDL mice, IL11 serum concentration was higher than SHAM control, but the experimentally induced elevation was effectively rescued after DZNep-treatment (Figure 8F). These data highlight that EZH2 inhibition by DZNep could diminish IL11 expression via targeting its upstream TGFβ signaling, suggesting IL11 as a crucial profibrotic factor in the liver beyond cardiovascular and pulmonary systems.

In addition to acting as the predominant contributor to hepatic myofibroblasts, HSCs also regulate liver inflammation and exert hepatoprotection through their own gene expression and interplay with other liver cells 51. Notably, in CCl_4_ and BDL mice, we found that DZNep administration alleviated liver injury and correlated with altered expression of interleukin genes in HSCs that play roles in inflammatory responses and fibrogenesis, among which, *Il10* was further characterized as direct target of EZH2. In the liver, IL10 and its receptor IL10Rα can be produced by HSCs and Kupffer cells. Their binding activates IL10Rα-STAT3 pathway, induces IL10 response genes expression, and thereby modulates liver inflammation and fibrosis. IL10 is very low in primary HSCs but is significantly increased during activation 38. *Il10* knockout mice or treatment with anit-IL10 antibody is associated with severe fibrosis 37, while its transgenic expression in liver alleviates fibrosis 52. In humans, IL10 treatment normalizes serum ALT level, improves liver histology and reduces liver fibrosis in patients with chronic hepatitis C 53, and the high IL10 production haplotype of its gene promoter is associated with reduced susceptibility to liver cirrhosis 54. Thus the protective role of DZNep against liver injury might be associated with the epigenetic regulation of *Il10* by EZH2. Interestingly, it was reported that GSK-J4 administration of macrophages and natural killer cells reduce IL10 expression 25, 55, raising the possibility that *IL10* may be among the common target genes regulated by both EZH2 and JMJD3.

Another notable aspect of this study is that EZH2 and JMJD3 epigenetically modulate HSCs phenotypes through regulating *CDKN1A* and *GADD45*. As a potent cyclin-dependent kinase inhibitor, CDKN1A, also known as p21, regulates cell progression, inhibits proliferation and induces senescence of HSCs, which facilitates the resolution of liver fibrosis 56, 57. *CDKN1A* polymorphisms that are associated with decreased p21 expression predispose to liver fibrosis and idiopathic pulmonary fibrosis 58, 59. GADD45A and GADD45B are members of the growth arrest and DNA damage-inducible 45 families that play key roles in growth suppression, DNA repair and apoptosis. Hepatic expression of GADD45A is suppressed in CCl_4_ mice, while its overexpression in HSCs inhibits expression of ECM proteins and α-SMA 60. *Gadd45a*-null mice with nonalcoholic steatohepatitis show more severe hepatic fibrosis and inflammation 61. The increased expressions of CDKN1A and GADD45 in HSCs are associated with inhibited HSCs activation and ameliorated liver fibrosis in rats treated with PPARγ agonist or histone deacetylase inhibitor 62, 63. It was reported that EZH2 regulates cellular senescence through epigenetically repressing the expression of p21 in cancer cells 64. In alike manner, JMJD3 can epigenetically upregulate* Gadd45a*, promote cell senescence and thus inhibit iPSCs reprogramming 65. The present study also adds *Cdkn1a* and *Gadd45* genes to the epigenetic regulatory network of EZH2 and JMJD3 in HSCs biology and hepatic fibrogenesis.

Due to the established oncogenic and pro-fibrotic functions of EZH2, its pharmacologic intervention with inhibitors including DZNep has been proposed as promising therapy for neoplastic and fibrotic disorders. EZH2 inhibition by DZNep as an epigenetic drug shows anti-proliferative, pro-apoptotic effects in cancer cells 66, and attenuates TGFβ-dependent HSCs activation 46. A few responsive signaling pathways and target genes have been shown to be associated with these therapeutic effects in animals 11, 13, 16, 48, 66. However, DZNep acts as a broad specificity inhibitor of histone methyltransferase that suppresses both repressive and active histone methylation marks 10, 67. In certain circumstances, EZH2 also has noncanonical non-methyltransferase functions that affect transcription and translation 68, 69. Thus we should cautiously point out that the gene signature of HSCs associated with DZNep treatment may be affected by these potential confounding factors. Indeed, in transcriptomic profiling of cells and tissues from experimentally diseased animals with DZNep treatment, we and other groups observed strikingly differential percent of DEGs were upregulated 10, 12. In this study, we also measured the *in vitro* effects of GSK126, a highly selective inhibitor of EZH2 methyltransferase activity, on the cellular phenotypes of HSCs. The immediate sharp reduction in cell viability associated with GSK126 treatment of mouse JS1 or human LX-2 cells suggests its cytotoxicity towards HSCs. We here clearly uncover a few important fibrosis related genes,* Bambi*, *Cdkn1a*, *Gadd45a*, *Gadd45b* and *Il10*, as direct epigenetic targets for the H3K27 methyltransferase activity of EZH2, their reactivation by DZNep treatment could, in part at least, account for the beneficial effects of DZNep against HSCs activation and liver fibrogenesis. Given that these target genes encode pivotal regulators for TGFβ signaling, cellular apoptosis and senescence, their epigenetic regulation by EZH2 may have relevance in fibrogenesis of other organs beyond liver, which shed more light into the underlying mechanisms of DZNep therapy against tissue fibrogenesis and tumorigenesis.

Compared with the well-defined oncogenic and pro-fibrotic functions of EZH2, JMJD3 is a double-edged sword with complex and somehow opposite functions in different context of disease, tissue and cell type. Accumulating evidence have shown that JMJD3 plays pro-inflammatory or anti-inflammatory in immune diseases, and carcinogenic or tumor suppressive roles in various types of cancer, which is associated with the histone demythylase activity-dependent epigenetic regulation and its noncanonical noncatalytic functions 23, 65. As JMJD3 stimulates a wide range of genes implicated in inflammation, immunity, development, senescence and carcinogenesis, its expression in normal conditions is usually in low level in the organization, just as its undetectable expression in the tested hepatic cell lines in this study, its activation is an important host response against environmental and cellular stress. As a JMJD3 specific small-molecular inhibitor, GSK-4 has been used in selective pharmacological intervention of JMJD3 in a few mouse disease models. In line with this scenario, the upregulation and pro-fibrotic role of JMJD3 in SSc, cardiac fibrosis and chronic cystitis induced bladder fibrosis have been recently reported 26, 27, 70. However, we here documented that JMJD3 was relatively highly expressed in rat primary HSCs but was rapidly silenced during their activation. The cellular phenotype of GSK-4 treatment and JMJD3 ectopic expression suggest it as a pivotal negative regulator for HSC activation, which could be ascribed to, in part at least, as discussed above, its epigenetic regulation towards target genes including *Bambi*, *Cdkn1a*, *Gadd45a* and *Gadd45b* that regulate HSCs activation, apoptosis and senescence. Although we did not found clear correlation between *JMJD3* expression and fibrosis staging in the cohort of liver fibrosis patients 29, a recent study showed that JMJD3 promotes hepatic autophagy by epigenetically upregulating global autophagy-network genes, the hepatic expression of JMJD3 and these key autophagy genes was substantially decreased in non-alcoholic fatty liver disease (NAFLD) patients (both simple steatosis and advanced NASH-fibrosis patients) as compared to normal subjects 71. We should acknowledge that this study has limitations and only provided, for the first time, *in vitro* evidence to support JMJD3 as key negative regulator against HSCs activation, HSCs-targeted *Jmjd3* transgene *in vivo* investigation in mouse models with adeno-associated viral vector, which is enabled to host large gene sequences like* Jmjd3* 72, is highly warranted to further elucidate the function of JMJD3 in liver fibrosis and its possible translational application for epigenetic gene therapy.

In summary, through pharmacological and genetic manipulations in HSCs and mouse liver fibrosis models and transcriptomic profiling of HSCs, we demonstrate that H3K27 methyltransferase EZH2 and demethylase JMJD3 regulate *Bambi*, *Cdkn1a*, *Gadd45a* and *Gadd45b* genes in HSCs, which is functionally associated with the antagonistic roles of EZH2 and JMJD3 in HSCs activation, and with the anti-proliferative, pro-apoptotic and anti-fibrotic benefits of EZH2 inhibitor DZNep treatment in both HSCs and mice. This study thus provides new mechanistic insight into the epigenetic modulation of EZH2 and JMJD3 in HSCs biology and hepatic fibrogenesis.

## Methods

### Cell lines, plasmids and experimental animals

Mouse HSC cell line JS1 was provided by Professor Jinsheng Guo at the Division of Digestive Diseases, Zhongshan Hospital of Fudan University. Human HSC cell line LX-2 was provided by professor Lieming Xu at Shanghai University of Traditional Chinese Medicine. The JS1 and LX-2 HSC cell lines were originally established by the providers respectively, and had been ascertained for identity with short tandem repeat (STR) genotyping. We obtained from Addgene the mouse *Ezh2* retroviral vector, MSCVhygro-F-*Ezh2* (cat. no. 24926) and the enzymatically inactive mutation vector, MSCVhygro-F-*Ezh2*-F667I (cat. no. 24927) 73, the plasmid pCS2-*Jmjd3*-F (cat. no. 17440) containing full-length mouse *Jmjd3* cDNA, and hereby generated a demethylase activity defective *Jmjd3* mutant mutating histidine 1388 to alanine (H1388A) with point mutation method 74. SPF grade BALB/C mice and Sprague-Dawley (SD) rats were obtained from the Shanghai SLAC Laboratory Animal Co. LTD, housed and maintained in the department of Laboratory Animal Science of Shanghai Medical College Fudan University. All animals have received human care, the protocols for animal experiments were approved by the Animal Ethics Committee of Shanghai Medical College Fudan University (Permit Number: 20130227-068), and conform to the *Animal Research: Reporting of In vivo Experiments* (ARRIVE) guidelines.

### Primary HSCs isolation, purification and culture

The primary HSCs were isolated from adult normal male SD rats (weighting 200-250 g) and purified using the improved method of *in situ* pronase (Sigma Aldrich, P5247)-collagenase (Sigma Aldrich, C5138) perfusion, followed by OptiPrep density gradient centrifugation according to the manufacturer protocol (Axis-shield, Norway). We determined HSCs viability by the Trypan blue exclusion test. The fresh primary HSCs were cultured in DMEM with 20% FBS in 5% CO_2_ at 37℃. The detailed procedure was described in our recent work 20, 32.

### Pharmacological inhibition *in vitro* and* in vivo*

We used DZNep (Selleck, S7120) , GSK126 (Selleck, S7061) and GSK-J4 (Selleck, S7070) as inhibitors of EZH2 and JMJD3 respectively, which are with DMSO (Dimethyl Sulfoxide) solvent, and used DMSO as negative control. Rat primary HSCs were treated with DZNep (1 μM), GSK126 (5 or 10 μM) or GSK-J4 (5 μM). DZNep and GSK126 treatment were also conducted in JS1 and LX-2 cell lines. Experimentally diseased mice (as below) were administered with DZNep (1 mg/kg) through intraperitoneal injection twice a week. The H3K27me2/3 levels in HSCs and in liver tissue were analyzed by Western blot to ascertain the inhibitory effect.

### RNA interference *in vitro* and* in vivo*

We used small interfering RNA (siRNA), short hairpin RNA (shRNA) and artificial microRNA (amiRNA) (synthetized by Shanghai HuaGene Biotech Company with sequence or target sequence listed in Table S1) to silence* Ezh2* in primary rat HSCs, mouse JS1 cells and diseased mouse liver, respectively. siRNA and shRNA specifically targeting rat or murine *Ezh2* gene, as well as their negative controls, were designed using the Sigma-Aldrich database. Rat primary HSCs were transfected with the *Ezh2* siRNA using HG-transgene reagent (Health & Gene, Ningbo, China) for the assay of lipid drop detection. The *Ezh2* shRNAs and negative control shRNA were inserted into retroviral vector pMOK.1-puro respectively, packaged into 293T Cell and then stably transfected into JS1 cells. We further utilized amiRNA-based RNAi strategy for *Ezh2* silencing *in vivo*, which shows advantages over traditional approaches by replacing the sequence of mature miRNA in its pre-miRNA stem-loop with the designed shRNA that targets specific gene of interest, thus permitting the artificial hairpin embedded in natural miRNA backbone to be processed into effective miRNA by the same cellular miRNA biogenesis machinery as natural miRNAs 32. The amiRNA for *Ezh2* silencing was based on the structural backbone of miR-30a as loading vehicle, which is most widely used for amiRNA vector construction, and the above two valid shRNAs of *Ezh2* as targeting cargos. We thereby constructed the HSC-specific GFAP promoter-driven lentiviral amiRNA expression vectors for *Ezh2* silencing and prepared the recombined lentivirus stock for RNAi according to the protocol described in detail in our recent work 32. We stably transfected JS1 cells with the pLenti-amiRNAs and 48 hours later analyzed EZH2 expression with Western blot to validate the gene silencing efficiency. In experimentally diseased mice, we transduced the pLenti-amiRNA virus particles into the liver through tail-vein injection (2×10^7^ infectious units/ml virus titer one time, every 5 days in one month), and analyzed the phenotypic effects on liver fibrosis in humanely sacrificed animals.

### Ectopic gene expression *in vitro*

The above wild and mutant *Jmjd3* gene cDNA were cloned into adenoviral expression vector (Vigene Bioscience, China). The above wild and mutant *Ezh2* retroviral vectors were packaged into 293T cells. Rat primary HSCs and mouse JS1 cells were infected with the adenovirus containing mouse *Jmjd3* and the retrovirus containing mouse *Ezh2* respectively with the concentration of 2×10^6^ infectious units/ml virus titer, and their ectopic expression in HSCs was determined by RT-qPCR and Western blot.

### RT-qPCR and Western blot

The total RNAs of cells or liver tissues were extracted using TRIzol reagent (Invitrogen, 15596-026) and reversely transcribed to cDNAs using Prime Script RT reagent kit (Takara, RR037A). The transcriptional expression of genes was determined by relative quantitative PCRs using SYBR Premix Ex Tap kit (Takara, DRR420A) and was analyzed with 2^-ΔΔ*Ct*^ method with β-actin as inner control. The RT-qPCR primers are listed in Table S2. The total proteins of cells or liver tissue were subjected to Western blot with primary antibodies for proteins and secondary antibodies (Table S3). The relative expression of proteins was determined by gray level of target protein band, which was determined by Image J software, as compare with that of inner control β-Actin. Gene and protein expression analysis was performed in triplicate for each sample, and statistical significance was evaluated basing on three independent experiments.

### Analyses of cell proliferation, cycle, apoptosis and senescence

JS1 and LX-2 cells with DZNep, GSK126 or DMSO treatment were cultured and analyzed for these cellular phenotypes. For proliferation assay: The cells were cultured in 96 well plates, each hole inoculated 2000 cells and 10 repeats were set for every treatment. In the following 1, 3 and 5 culture day, cellular activity was measured by CCK-8 kit (Dojindo, CK04-3000T), then the cell growth curve was draw according to the absorbance of cells (OD_450_). For cell cycle and apoptosis assay: After 48 hours culture, the cells were collected, either stained with propidium iodide (PI) or double stained with Annexin-V-FITC/PI, and were subjected to flow cytometer analysis. For DNA breakage induced cellular apoptosis assay: The cells were fixed and stained by FITC-dUTP using One Step TUNEL Apoptosis Assay kit (Beyotime Biotechnology, C1086), then the apoptotic cells were detected with green fluorescence. For cellular senescence assay: After 96 hours culture, the cells were lysed, and total protein concentration of cell lysates was determined with BCA protein assay (Pierce, A53225). The senescence-associated β-galactosidase (SA-β-Gal) activity of cell lysates was measured with cellular senescence assay kit (Cell Biolabs, CBA 231). The value of each SA-β-Gal activity was standardized by its total protein concentration. Each data was measured from triple independent experiments.

### Liver fibrosis *in vivo* models

We investigated the disease relevance of EZH2 in two conventional murine models of experimental liver fibrosis, carbon tetrachloride-induced toxic injury (CCl_4_), bile duct ligation-induced cholestatic injury (BDL). In normal BALB/C mice (each body weighs about 20 g and five weeks old), liver fibrosis was induced by intraperitoneal injection of CCl_4_ diluent (CCl_4_: olive oil is 1:4; 2.5 ml/kg body weight every 3 days). Normal mice were treated with DMSO. BDL model was induced by ligating the common bile duct under surgical operation. In both models, EZH2 inhibition was accomplished by intraperitoneal injection DZNep or DMSO as control (as above). Sham-operated animals were used as control. In CCl_4_ model, HSCs-specific *Ezh2* silencing *in vivo* was accomplished by the amiRNA-based RNAi (as above). After one month for CCl_4_ model and 14 days for BDL model, the mice were humanely sacrificed and serum and liver samples were prepared. The serum alanine aminotransferase (ALT) and aspartate aminotransferase (AST) enzyme activity were measured by automatic biochemistry analyzer (Beckman Coulter). Serum concentrations of IL10 and IL11 were measured with ELISA (YuanYe Biotechnology, China, CK-E30651R and CK-E30539R).

### Masson's trichrome staining, immunohistochemistry and hydroxyproline assay

The liver tissue blocks were fixed by 4% paraformaldehyde, and then embedded into paraffin blocks. The liver tissue slices of 7 μm thick were stained using Masson's trichrome staining kit (Sigma, HT15-1KT) or subjected to immunohistochemistry. The slices were taken photos at 200× magnification from six random fields for each one. Collagen secretion was evaluated by the blue area of Masson trichrome staining, and α-SMA expression was calculated by the integrated optical density (IOD) of positive area of immunohistochemistry slices, both of them were analyzed with Image-pro plus soft. The liver fibrosis level was determined as the mean of six random different fields of each section. The content of hydroxyproline in liver tissues and serum was measured by hydroxyproline assay kit according to manufacturer's protocol (Cell Biolabs, STA-675).

### RNA sequencing and bioinformatics analysis

The primary rat HSCs were treated with 1 μM DZNep (diluted with DMSO) or DMSO as negative control, each treatment was conducted three replicates. 48 hours later, cells were harvested and total RNA was isolated. RNA-seq libraries were prepared and sequenced using HiSeq3000 (Ribo Bio Co., Ltd. Guangzhou). The genes expression profiling of rat primary HSCs with or without DZNep treatment were analyzed by PolyA-seq. The raw data were trimmed, filtered and qualified using FASTX (http://hannonlab.cshl.edu/fastx_toolkit/), clean data was aligned to rat reference genome (rn5) using TopHat2. The distributions of reads on the genome were analyzed, and the locations of reads on the exon, intronic and intergenic areas were also annotated. The expression level of every gene was evaluated by RPKM method (expected number of reads per kilobase of transcript sequence per million base pairs sequenced). Then differential expression genes (DEGs) in the HSCs with or without DZNep treatment were screened by DESeq, according to the criteria of 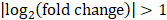 and *q*-value < 0.05. The *q*-value was an adjusted *p*-value for false discovery rate (FDR). The expression profiles of DEGs between samples were analyzed by hierarchical clustering analysis. The DEGs were analyzed by the upstream regulator analysis (URA) of Ingenuity Pathway Analysis (IPA), to predict the relevant upstream regulators that are associated with the effect of DZNep on HSCs. *Log_2_(fold change)* was utilized to calculate the *Z*-score of each upstream regulator. Before uploading the quantified DEGs list to IPA, we replaced the positive and negative infinity values of *Log_2_(fold change)* by 8 and -10 respectively. The gene symbols of the uploaded DEGs were strictly mapped to genes and gene products of rat, while interactions were allowed to extend to which has been reported in human or mouse. We only used experimentally validated and high confident predicted evidences in IPA. 28,674 of all the 49,752 RNA-seq tracking ids were with RPKM > 0 at least in one batch. We used the R package ***fcro***s (version: 1.6.1) to get the average of rank values (*ri*) associated with those 28,674 tracking ids, and used 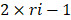 as the final rank scores in the pre-ranked gene list (*.rnk file). The pre-ranked gene list was used for Gene Set Enrichment Analysis (GSEA) through the WEB-based GEne SeT AnaLysis Toolkit (WebGestalt) 75. Organism “rat”, method “GSEA”, databases “pathway” and “geneontology” were chosen, and default parameters with top 10 *NES* results were assigned. All RNA-seq raw data for this study have been deposited in the NCBI Gene Expression Omnibus database (GSE121736).

### ChIP-qPCR

The primary rat HSCs were administrated with EZH2 inhibitor DZNep, DMSO, or were infected with adenovirus recombined with mouse *Jmjd3* gene (AD-*Jmjd3*) or AD-*GFP* as control. After four days treatment, these cells were harvested and sonicated by bioruptor (Diagenode, Belgium). By using chromatin immunoprecipitation (ChIP) assay kit (Upstate, cat no. 17-371), the supernatant of sonicated cells was co-immunoprecipitated with the anti-H3K27me3 antibody (Millipore, Germany, 07-449) or the anti-H3K27me2/me3 antibody (Active Motif, China, 39535) to assess the binding of EZH2 or JMJD3 respectively, and the mouse IgG was used as negative control. The enrichment of target gene fragment in DNA precipitate was analyzed by quantitative real-time PCR, and the primers for target genes were listed in Table S4. The fold enrichments of target genes between ChIP-DNA and input-DNA were determined by Δ*Ct*.

### Statistical analysis

The data were expressed as the means ± standard deviation of at least three independent experiments for every assay. The statistical significance was analyzed by Student's *t-*test. *P* < 0.05 were considered significant.

## Supplementary Material

Supplementary figures and tables.
