# Supplementary material for: Histone H3K27 methyltransferase EZH2 and demethylase JMJD3 regulate hepatic stellate cells activation and liver fibrosis
Source: Theranostics. 2021 Jan 1;11(1):361–78. doi: 10.7150/thno.46360 (PMC7681085; doi:10.7150/thno.46360)
Supplement: Supplementary file 1 — Supplementary figures and tables. [file thnov11p0361s1.pdf]

Histone H3K27 methyltransferase EZH2 and demethylase JMJD3 regulate hepatic stellate cells activation and liver fibrosis

Yan Jiang, Chan Xiang, Fan Zhong, Yang Zhang, Liyan Wang, Yuanyuan Zhao, Jiucun Wang, Chen Ding, Li Jin, Fuchu He, Haijian Wang

Supplementary Data

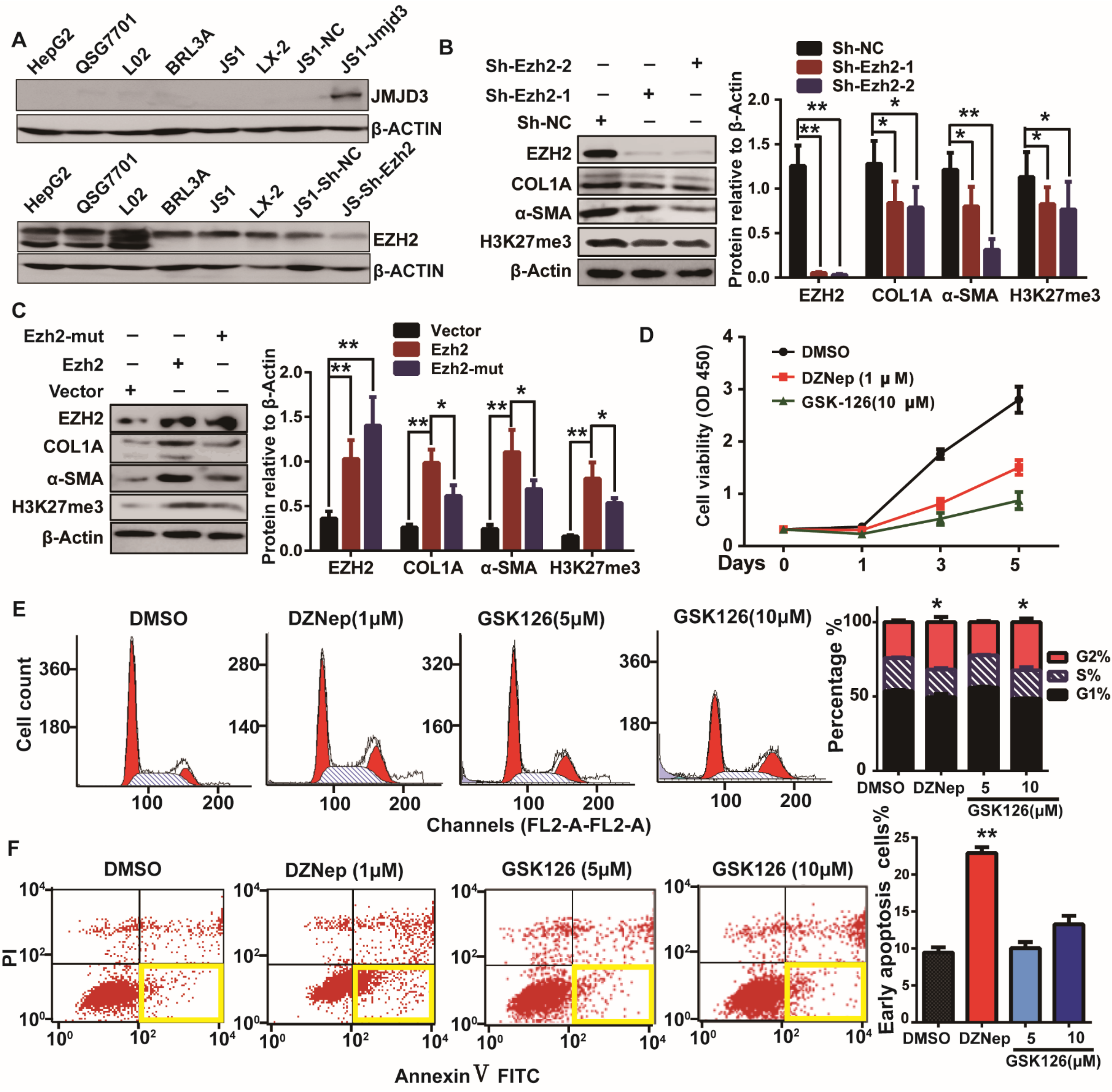

Supplementary Figure 1 EZH2 and JMJD3 expression in hepatic cell lines and functional role of EZH2 on cellular phenotypes of HSCs

EZH2 and JMJD3 expression in various hepatic cell lines were measured by Western blot (A). The effect of stable silencing (B) or stable overexpression (C) of EZH2 in mouse JS1 cells, which was based on retrovirally-expressed shRNA or *Ezh2* gene respectively, on the expression of EZH2, COL1A, α-SMA, β-ACTIN and H3K27me3 were determined by Western blot.

Human LX-2 cells were treated with DMSO, DZNep or GSK126 and then subjected to cell growth, cell cycle and apoptosis analysis. (D) The cellular growth curve was drawn according to cellular OD_{450} value measured by CCK-8 kit. (E) The cells in G1, S or G2/M phase were stained with PI and counted separately using flow cytometry. Cell cycle arrest was evaluated by calculating the percent of cells in each phase. (F) The apoptotic cells were determined by Annexin Ⅴ-PI double staining. The early apoptotic cells stained with high Annexin Ⅴ and low PI (in yellow frame) were counted using flow cytometry. Statistical significance was evaluated with Student's *t*-test in independent-samples, and $P < 0.05$ was considered as significant difference, * $P < 0.05$, ** $P < 0.01$.

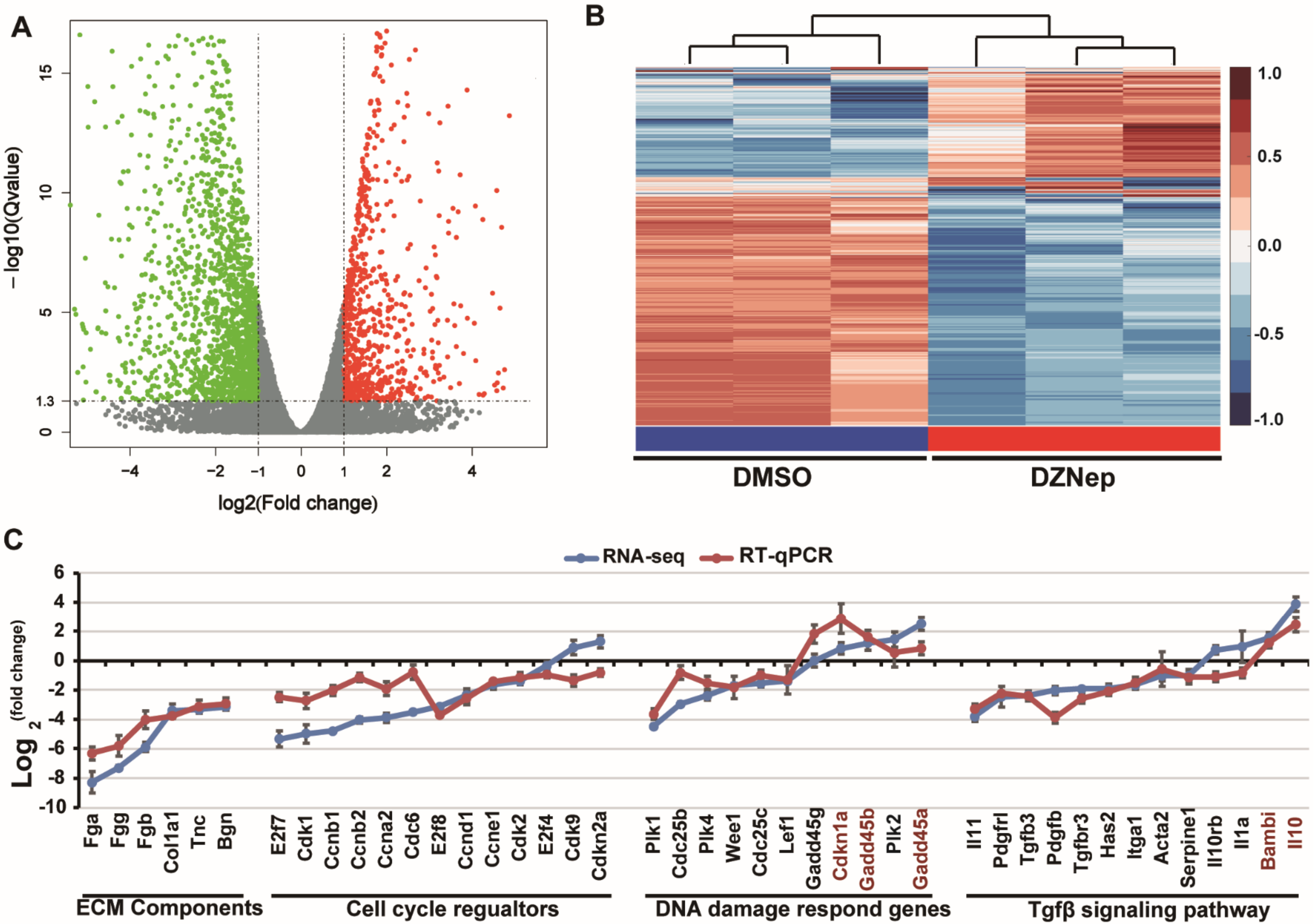
A
−log10(Qvalue)
log2(Fold change)
B
1.0
0.5
0.0
-0.5
-1.0
DMSO
DZNep
C
RNA-seq
RT-qPCR
Log 2 (fold change)
Fga
Fgg
Fgb
Col1a1
Tnc
Bgn
E2f7
Cdk1
Ccnb1
Ccnb2
Ccna2
Cdc6
E2f8
Ccnd1
Ccne1
Cdk2
E2f4
Cdk9
Cdkn2a
Plk1
Cdc25b
Plk4
Wee1
Cdc25c
Lef1
Gadd45g
Cdkn1a
Gadd45b
Plk2
Gadd45a
Il11
Pdgfrl
Tgfb3
Pdgfb
Tgfbr3
Has2
Itga1
Acta2
Serpine1
Il10rb
Il1a
Bambi
Il10
ECM Components
Cell cycle regualtors
DNA damage respond genes
Tgfβ signaling pathway

Supplementary Figure 2 The differential expression genes associated with DZNep treatment in rat primary HSCs were screened with RNA-seq and some of them were validated with RT-qPCR

(A) Volcano plot shows the significantly upregulated (red dots) and downregulated (green dots) genes associated with DZNep treatment in HSCs. (B) The heatmap shows the transcriptional value change of differential expression genes induced by DZNep. Hierarchical clustering analysis of the results of three treatment and three control experiments verified the biological replication. (C) The transcriptional values of 43 candidate differential expression genes that are related with fibrosis were also measured by RT-qPCR. We validated consistent transcriptional expression changes between the readouts of RNA-seq and RT-qPCR for 95% detected DEGs. Fold change was calculated as the ratio of the gene expression value in DNZeP-treatment cells to that in DMSO-treatment cells. The scatter diagram and trend line analyzed the consistency of the gene expression trend measured by RNA-seq and by RT-qPCR. Y-axis value denotes the $\log_2^{(\text{fold change})}$; X-axis presents the key differential expression genes involved in ECM components, cell cycle, DNA damage and response pathway and TGFβ signaling pathway.

Supplementary Table 1 : The target sequence of si-RNAs, sh-RNAs and amiRNAs for *Ezh2* silencing

Name	The target sequence (5′-3′)
Rat Ezh2-si-RNA1	TAGAGTCCTCATTGGTACT
Rat Ezh2-si-RNA2	ACGGCTCCTCTAACCATGT
Ezh2-si-NC	TTCTCCGAACGTGTCACGT
Mouse Ezh2-sh-RNA1	GCTGAAGCCTCCATGTTTAGA
Mouse Ezh2-sh-RNA2	GCACAAGTCATCCCGTTAAAG
Mouse Ezh2-sh-NC1	CCTAAGGTTAAGTCGCCCTCG
Mouse Ezh2-sh-NC2	TTCTCCGAACGTGTCACGT
amiRNA-Ezh2	CTCGAGGTCGACTAGGGATAACAGGGTAATTGTTTGAATGAGGCTTCAGTACTTTACAGAATCGTTGCTGAAGCCTCCATGTTTAGAGTGAAGCCACAGATGTATCTAAACATGGAGGCTTCAGCGCCTGCACATCTTGGAAACAGCTGGGATTACTTCTTCAGGTTAACCCAACAGAAGGCTCGAAAAGGTATATTGCTGTTGACAGTGAGCGCCGCACAAGTCATCCCGTTAAAGGTGAAGCCACAGATGTACTTTAACGGGATGACTTGTGCTGCCTACTGCCTCGTCTAGAAAGGGGCTACTTTAGGAGCAATTATCTTGTTTACTAAAACTGAATACCTTGCTATCTCTTTGAT ACA TTTTTTGGATCC
amiRNA-NC	CTCGAGGTCGACTAGGGATAACAGGGTAATTGTTTGAATGAGGCTTCAGTACTTTACAGAATCGTTCCTAAGGTTAAGTCGCCCTCGGTGAAGCCACAGATGTACGAGGGCGACTTAACCTTAGGGCCTGCACATCTTGGAAACAGCTGGGATTACTTCTTCAGGTTAACCCAACAGAAGGCTCGAAAAGGTATATTGCTGTTGACAGTGAGCGCCTTCTCCGAACGTGTCACGTGTGAAGCCACAGATGTAACGTGACACGTTCGGAGAATGCCTACTGCCTCGTCTAGAAAGGGGCTACTTTAGGAGCAATTATCTTGTTTACTAAAACTGAATACCTTGCTATCTCTTTGATACA TTTTTTGGATCC

Supplementary Table 2: Primers for RT-qPCR

Genes	Forward (5′-3′)	Reverse (5′-3′)
Rat Genes		
Actb	AGAGGGAAATCGTGCGTGACA	ACATCTGCTGGAAGGTGGACA
Bambi	CTGCTCACCAAAGGCGAGAT	GATGTCTGCTGTGCTTGCGA
Bgn	TCCCCAGGAACATTGACCAT	TGAGCAGCCCATCATCCAAG
Ccna2	CACGTACCTTAGGGAAATGG	CCAAATGCAGGGTCTCATTC
Ccnb1	TGAGCC TGAACCTGTTATGG	CCACCATCGTCTGCATCTAC
Ccnb2	GCTGGGCCAAGGAAAATGGA	TGCCTAGGGTCTGCCCATCA
Ccnd1	TCAAGTGTGACCCGGACTG	CACTACTTGGTGACTCCCGC
Ccne1	ATGTCCAAGTGGCCTACGTC	CTTTCTTTGCTTGGGCTTTG
Cdc25b	TCCCTGTGTCACGAGATTGAG	TCAACAGGGCCACCATAGTTTCT
Cdc25c	TGGTGATTTCTCAAAGGCGTG	GGGCTGATATACTTCAGATCCTGG
Cdc6	ACCACTCTCCGAATGTAAATCAC	ACGACAGACACTACTGTAGGC
Cdk1	TGGCCAGTTCATGGATTC	GCCGAAATCTGCCAGTTTG
Cdk2	CACTTAACCCGACTTCCAG	TTCCCTCAACACGGTAAC
Cdk9	GAATGCCCGTTTTGCGATGA	TGATGGGGAACCCCTCCTTC
Cdkn1a	AGACCAGCCTAACAGATTTCTATC	GACACACTGAATGAAGGCTAAGG
Cdkn2a	TCGTACCCCGATACAGGTGAT	TGTCTAGGAAGCCCTCCCG
Clo1a1	TTCACCTACAGCACGCTTGTG	GATGACTGTCTTGCCCCAAGTT
E2f1	AGCGCCTGGCCTATGTGACCTG	TCGATGGGGCCTTGTTTGCTCTTA
E2f4	ATTGCAGTGAGTGGTAGCCC	TTTGGGGAGATCCAGAACGC
E2f7	GCCTTCAAATGGATCGGGC	GGAATAGGCTGGCCCTTGTTTTC
E2f8	CTCCCCAATTGCAGGTGTGA	AGACGTCGGGGAGACCATAA
Fga	GGCCTATAAAACAGAACAGTGTC	GGGCATTTGTGGTTCCAGTC
Fgb	GCTCAGACGGAATACTGCCA	TATGACCGTCCATCCTCCGT
Fgg	CCAAACAGGTTGGAGACATGTAA	ATCGCCAGCATAAAACTGCT
Gadd45a	TGCTCAGCAAGGCTCGGAGT	GTTGCTGACCCGCAGGATGT
Gadd45b	ACTGATGAATGTGGACCCCG	CATGCCTGATACCCTGACGA
Gadd45g	AACTTGCTGTTCGTGGATCG	ACATTGTCAGGGTCCACATTC
Has2	TCAGACACCATGCTTGACCC	AGAGGACCGCTTATGCACTG
Il10	AAGGGTTACTTGGGTTGCCA	AAATCGATGACAGCGTCGCA
Il10rb	TGGTACTTCCAAGACCGCTG	CGATAATGGTGTCTTCCACGG
Il11	CAGCTCTTGATGTCTCGCCT	TTTAACAACAGCAGGCCCCG
Il1a	CCTGTGTTGCTGAAGGAGATTC	CTATCATGGAGGGCAGTCCC
Itga1	TGATGACGCTCTGCCAAACT	CACCACTGTCCTGGTGTTGT
Lef1	GGGACACTTCCATGTCCAGG	AGGCTTCACGTGCATTAGGT
Mki67	GCAGCTTCTACCAAGAGGCA	GGGGCTTGGCTGTTTTTCAG
Pai1	CGTCTTCCTCCACAGCCATT	GCTGGCCCATGAAGAGGATT
Pdgfb	GACTCCGTAGACGAAGATGGG	CAGGAAGTTGGCATTGGTGC
Pdgfbr1	AATGACCACGGCGATGAGAAAG	AGGACAGAGGGCGTCGGATAA
Plk1	TTGAGGACAGCGACTTTGTG	GCGCCTTCCTCCTTTTGT
Plk2	CACCACCATCATCACCATTC	TCGTAACACTTTGCAAATCCA
Plk4	GCCAATGAGGGTCACCGTA	CGCACTATTCGCGCTCAATC
Tgfb3	TACCTCCGCAGCTCAGACAC	TTCTGCCAACATAGTACAAG

Tgfbr3	CGGCTTTGGAAAAGAGAGTG	CAGGAGGAATGGTGTGGACT
Tnc	CAGCTACCGACGGGATCTTC	TTCCGGTTCAGCTTCTGTGG
Wee1	CTACTTTCTGGGCAGCTCGT	GGAAAGCAAACTCTTGGGAGTG
Mouse Genes		
Acta2	CGGGAGAAAATGACCCAGATT	AGGGACAGCACAGCCTGAATAG
Actb	GGCTCCTAGCACCATGAAGA	AGGGTGTAAAACGCAGCTCAG
Bambi	CGAAGCCTCAGGACAAGGAAA	GCATTCGCAAGGCCAACATA
Cdkn1a	GAATAAAAGGTGCCACAGGC	CAAAGTTCCACCGTTCTCGG
Col1a1	GGAGAGTACTGGATCGACCCTAAC	ACACAGGTCTGACCTGTCTCCAT
Mki67	TTGGTGGACATCTAAGACCTGA	GGGCCGTTCCTTGATGATTGT
Mmp2	GTTCAACGGTCGGGAATACA	GCCATACTTGCCATCCTTCT
Human Gene		
MKI67	TTACCGGGCGGAGGTATGAA	GCTGGCTCCTGTTCACGTAT

Supplementary Table 3: Antibodies used in this study

Primary antibody	Catalog No.	Company (country)
Anti-β-ACTIN (Mouse)	A00702	Gen Script (USA)
Anti-EZH2 (Rabbit)	21800-1-AP	Proteintech (USA)
Anti-JMJD3 (Rabbit)	AP1022a	Abgent (USA)
Anti-α-SMA (Rabbit)	Ab5694	Abcam (China)
Anti-COL1A (Mouse)	Ab6308	Abcam (China)
Anti-BAMBI (Rabbit)	Ab203070	Abcam (China)
Anti-CDKN1A (Rabbit)	Ab109199	Abcam (China)
Anti-GADD45B (Rabbit)	Ab205252	Abcam (China)
Anti-IL10 (Rat)	Ab33471	Abcam (China)
Anti-IL11(Rat)	sc-133084	Santa cruz (China)
Anti-H3K27me2 (Rabbit)	Ab24684	Abcam (China)
Anti-H3K27me3 (Rabbit)	07-449	Millipore (Germany)
Anti-H3K27me2/me3 (Mouse)	39535	Active Motif (China)
Anit-Histone3 (Mouse)	61475	Active Motif (China)
Secondary antibody		
Mouse anti-rabbit	211-032-171	Jackson Immuno Reseach
Goat anti-mouse	115-005-205	Jackson Immuno Reseach
Goat anti-rat	112-225-143	Jackson Immuno Reseach

Supplementary Table 4: Primers for ChIP-qPCR

Rat Genes [a]	Forward (5′-3′)	Reverse (5′-3′)
Bambi (-1258)	GTGTGTTTGCCTGCGATTGT	TTCTCCTGGGTAGACTGGGG
Bambi (-347)	CAGCCAATCGGAGAGTGGAG	CCGGAGTTAGACGTATCCG
Bambi (+2951)	GCTTTTGGAGCACTTCCGTC	CAGTGAGCGGCATCACAGTA
Cdkn1a (-465)	CACTTCCTCTCCCCTCCTGA	AGAGAAGGACAGCCAGGGAT
Cdkn1a (-221)	AGCCAGCTTTCTGGCTTTCA	GTTAGCAGGAACTCGGGCTT
Cdkn1a (+494)	TTGGACATCCTGTGCTGGTC	TCGACAGCCTGGTTCTGTTC
Gadd45a (-707)	ACCAGCTTACAAGGAGTGGG	TGATGGCACAGTACCGAGTT
Gadd45a (-374)	TGAGCTTGGCTCGTTAGACA	CTGCCCCACTCCTTGTAAGC
Gadd45a (+635)	CTGGCAGAGCTGTTGCTACT	TCTTACCGTCACCAGCACAC
Gadd45b (-387)	TCTCCCCGAAAGTTCAAGCC	GACTGCCAGCGAATCGAGAG
Gadd45b (+92)	GAAAGTAAGTCCCACCGCCT	TCAGTCACACTTCACAGCGG
Gadd45b (+260)	GACAACGCGGTTCAGAAGTG	GTCACCGCCTGCATCCTAAA
Il10 (-695)	TCCCGTCAAAGAGTGTTGGG	GGGTTACCATACTGGAGCCG
Il10 (+76)	TGCTAAGGTGACCTCCTGGT	CCTGGGTTGAATGTCCGCTA
Il10 (+1767)	GGTGCCGTGGCTTTCAAAAA	TGGAAGGATGGACTGTTGCC
Il11(-704)	ACACCCTCAGCTCCTCAGTT	GAACACTGGGACAGGGATGG
Il11(-332)	GAGCCTTGTGTCTGTCCCAG	AGGGCACGGAAGGAAAAGTT
Il11(+465)	GACGACCACGAACTCCCAAC	GTCCCCTCTAGCTGTGCCTA

[a] The 5′ endpoints of PCR products are positioned as upstream (-) or downstream (+) from transcription start sites.
